# Membrane-bound IL-15 co-expression powers a potent and persistent CD70-targeted TRuC T-cell therapy

**DOI:** 10.3389/fimmu.2025.1609658

**Published:** 2025-05-30

**Authors:** Lindsay Webb, Michael Lofgren, Troy Patterson, Amy Watt, Jason Lajoie, Adam Zieba, Michelle Fleury, Erica Liu, Jian Ding, Robert Tighe

**Affiliations:** ^1^ TCR^2^ Therapeutics, Inc., Cambridge, MA, United States; ^2^ Adaptimmune, Cambridge, MA, United States

**Keywords:** TRuC T-cell therapy, CD70, IL-15, renal cell carcinoma, solid tumor, T-cell immunotherapy, fratricide resistant, persistence

## Abstract

**Introduction:**

Although T-cell immunotherapies have been effective in the treatment of hematological malignancies, solid tumors have proven challenging due to the immunosuppressive microenvironment and lack of viable target antigens. The immune checkpoint ligand CD70, overexpressed in several solid tumors, yet with limited expression in healthy tissue, has emerged as a promising immunotherapeutic target.

**Method:**

This study describes the generation and preclinical characterization of ADP-520, a high-affinity, fratricide-resistant, CD70-targeted T-cell receptor fusion construct (TRuC) T-cell therapy enhanced with constitutively expressed mbIL-15, a membrane-bound fusion protein comprising interleukin-15 (IL-15) linked to full-length IL-15 receptor-alpha. The phenotypic distribution, expansion and persistence of ADP-520 TRuC T cells were measured *in vitro* under autonomous and antigen-dependent conditions, with the contributions of TCR and IL-15 signaling pathways ascertained using inhibition assays. Chronic antigen stimulation was used to evaluate exhaustion-resistance, while anti-tumor potency was explored both *in vitro* and *in vivo*.

**Results:**

ADP-520 was found to have potent and antigen-specific activity against hematological and solid CD70-expressing tumors, without apparent fratricide or killing of bystander T cells despite CD70 expression by activated lymphocytes. Engineered co-expression of mbIL-15 augmented antigen-dependent expansion through pro-survival effects and enrichment of an early memory T-cell phenotype, thus enhancing tumor-autonomous, exogenous cytokine-free persistence and bolstering exhaustion resistance during chronic stimulation. mbIL-15 co-expression also enhanced intratumoral T-cell infiltration *in vivo* for potent and persistent antitumor efficacy.

**Discussion:**

These findings characterize ADP-520 as a first-in-class, CD70-targeted, fratricide-resistant autologous TRuC T-cell therapy leveraging native TCR signaling combined with constitutive IL-15 signaling to impart T cells with enhanced persistence, tumor penetration, and antitumor efficacy. This makes ADP-520 a promising cell immunotherapy candidate for clinical development, with the potential to overcome hurdles intrinsic to the treatment of solid tumors.

## 1 Introduction

Adoptive cell therapies (ACTs) take advantage of genetic engineering of the T-cell receptor (TCR) to convey antitumor function. Two main categories of engineered ACT T cells are being developed: chimeric antigen receptor (CAR) and engineered TCR T cells. CARs are formed by pairing an extracellular tumor antigen-binding domain to a membrane-spanning fusion protein containing an intracellular signaling domain, the latter most often comprising the TCR cluster of differentiation 3-zeta (CD3ζ) signaling chain fused to one or more co-stimulatory domains (1, 2). CAR T cells have demonstrated clinical safety and efficacy against hematological cancers, with six different therapies currently approved by the US Food and Drug Administration (FDA) across a range of indications (3). Nevertheless, poor tumor penetration, CAR T-cell exhaustion, an immunosuppressive tumor environment and a lack of viable target antigens have confounded CAR T-cell efficacy against solid tumors (4). T-cell activation by CAR domains does not recapitulate the natural signaling cascade of the TCR and has been implicated in the lack of solid tumor efficacy (5–7). In contrast, engineered TCR T-cell therapies employ the natural TCR signaling complex and evoke an effector response upon recognizing tumor peptide antigens presented by the human leukocyte antigen (HLA) system. These peptide-HLA (pHLA) targeting ACTs, such as those targeting the New York esophageal squamous cell carcinoma-1 (NY-ESO-1) or melanoma-associated antigen A4 (MAGE-A4) pHLAs, have demonstrated clinical efficacy against solid tumors, with the MAGE-A4 TCR T-cell therapy afamitresgene autoleucel recently receiving regulatory approval (8). However, these approaches require HLA matching and remain challenged by tumor-associated HLA downregulation (9–11).

Combining the advantages of both CAR and TCR T-cell ACT platforms, we developed the TCR fusion construct (TRuC) platform comprising a tumor-antigen binding moiety fused via a flexible linker to full length CD3ε or CD3γ subunits (6). The efficient assimilation of TRuCs into the endogenous TCR complex provide TRuC-TCRs with HLA-independent access to the natural TCR signaling and activation pathways, thus addressing a potential shortcoming of CAR-T while also bypassing HLA-dependent factors limiting TCR T-cell ACTs in solid tumor settings (7). Indeed, in preclinical studies the mesothelin (MSLN)-specific TRuC T-cell therapy gavocabtagene autoleucel (‘gavo-cel’, formerly TC-210) exhibited potential functional advantages over analogous MSLN-targeted CAR T cells (7). Moreover, gavo-cel demonstrated tolerability and anti-tumor activity in a Phase 1/2 trial in patients with MSLN-positive mesothelioma and ovarian cancer (NCT03907852) (12). A second-generation variant of gavo-cel called TC-510 was subsequently developed by incorporating a costimulatory programmed cell death protein 1 (PD-1):CD28 switch receptor to enhance signaling, promote proliferation and reduce T-cell exhaustion, and demonstrated tumor regression but apparent target-related toxicity in a Phase 1/2 clinical trial in MSLN-expressing solid tumors (NCT05451849) (13).

T-cell persistence remains a significant hindrance to the efficacy of ACT in solid tumors (14). One strategy for overcoming this challenge involves further engineering ACTs with T-cell activating cytokines. This approach improves anti-tumor activity by enhancing T-cell effector function and increasing T-cell persistence in the tumor microenvironment (2, 15). Interleukin (IL)-15 is known to promote the survival and expansion of T cells (16, 17), and thus IL-15 armoring is conducive to the long-lived persistence of T-cell ACTs (15). For example, incorporation of a membrane-bound IL-15 (mbIL-15) into CAR T-cell platforms has been shown to enhance the survival and function of T-cell therapies in a hostile tumor microenvironment (15, 18).

Tumor penetration is another hurdle to overcome in the treatment of solid tumors; however, some are immune infiltrated and thus suitable candidates for solid-tumor targeted ACT. Renal cell carcinomas (RCCs) are known to be highly infiltrated by CD8^+^ T cells, a phenomenon associated with an improved response to immunotherapy (19). Nevertheless, RCC continues to have high unmet needs with poor five-year survival rates (20). RCC is marked by high expression of the immune checkpoint ligand CD70, a target otherwise poorly expressed in normal tissue with the exception of transient expression in dendritic cell subsets and antigen-activated lymphocytes (21–23). CD70, signaling through its T-cell costimulatory receptor CD27, engages diverse functions in T-cell immunoregulation (22–26), while CD70–CD27 dysregulation has been associated with tumor progression (23). Several CD70-targeted immunotherapies are currently in clinical development, including two allogeneic CD70 CAR T-cell therapies being trialed in RCC: CTX130 and ALLO-316. Fratricide is a chief concern for CD70-targeted T-cell therapies, but this has been overcome through knockout of CD70 in the case of CTX130 (27, 28) and selection of fratricide-resistant development candidates wherein CD70 is masked via in cis CAR binding in the case of ALLO-316 (29).

Herein we describe the generation and preclinical characterization of ADP-520 (formerly TC-520), a novel CD70-targeted TRuC T-cell therapy, with an enhanced memory phenotype, improved expansion, low tonic activation and high on-target cytotoxicity. ADP-520 is a fratricide-resistant, mbIL-15 armored TRuC T-cell platform that exhibits potent and antigen-specific anticancer activity *in vitro* and *in vivo*, with an improved efficacy and persistence profile conducive to future clinical applications in the treatment of solid tumors such as RCC, or even hematologic malignancies such as acute myeloid leukemia (AML).

## 2 Materials and methods

### 2.1 Cell lines

The RCC cell lines 786-O (RRID: CVCL_1051) and ACHN (RRID: CVCL_1067), AML cell lines THP-1 (RRID: CVCL_0006) and MOLM-13 (RRID: CVCL_2119), and NSCLC cell line NCI-H1975 (RRID: CVCL_1511), were acquired from ATCC and cultured in RPMI medium (Corning, Manassas, VA) with 10% heat-inactivated fetal bovine serum (FBS; VWR, Radnor, PA). T-cell lymphoma cell lines, HuT 78 (RRID: CVCL_0337) and MJ (RRID: CVCL_1414), were acquired from ATCC and cultured in 80% IMDM (Corning) with 20% FBS. All cell lines were stably transduced with a lentivirus containing the luciferase gene and neomycin resistance and were selected with geneticin (Gibco, Grand Island, NY).

### 2.2 Antibody discovery

The C10 scFv clone was discovered by serial biopanning enrichment of a fully human naïve scFv phage display library (AbCheck, Czech Republic) against recombinant biotinylated human CD70 (Acro Bio, Japan), human IgG1 Fc-fused CD70 antigen (Acro Bio) and CHO cells expressing human CD70 (BPS Bioscience, San Diego, CA). Enriched clones were selected by flow cytometric staining of CD70-expressing CHO and/or human RERF-LC-KJ (RRID: CVCL_1654) cells and binding to recombinant His-tagged CD70 in an enzyme-linked immunosorbent assay (ELISA).

The 15F6 VHH clone was obtained by biopanning a phage display library generated from an alpaca immunized with a panel of human tumor cell lines, including KOPN-8 (RRID: CVCL_1866) and JVM-3 (RRID: CVCL_1320), that surface express human CD70. Anti-CD70 VHH clones were enriched during three rounds of immune phage library positive selection against recombinant human CD70 antigen, validated by fluorescence-activated cell sorting against human CD70 RCC cell lines and recombinant antigen via ELISA.

Unique enriched anti-CD70 clones were identified by HCDR3 clonotyping after Sanger sequencing and subcloned into a mammalian IgG1 Fc fusion expression plasmid for expression using an HEK system and purification using Protein A/G beads. Carterra LSA Surface Plasmon Resonance and OctetRed96 Biolayer Interferometry instruments were used to confirm CD70 binding competition against human CD27 and epitope bin grouping.

### 2.3 Generation of CD70-targeted TRuC T cells and ADP-520 TRuC T cells

The CD70-targeted ϵ-TRuCs were generated by tethering the sequence of the CD70-specific scFv or VHH to the TCR CD3ϵ subunit via a flexible linker (GGGGSx3). ADP-520 TRuCs were generated by incorporating an mbIL-15 sequence into the anti-CD70 scFv (C10)-CD3ϵ construct following a 2A sequence. The mbIL-15 construct was generated by fusing human IL-15 to the full-length IL-15Rα sequence via a flexible linker. The constructs were cloned into lentiviral transfer plasmids for vector generation.

Lentiviruses were prepared by transient transfection of Expi293 suspension cells with packaging plasmids and the TRuC lentiviral transfer plasmids, as previously described (6). Cells were centrifuged 48 hours after transfection and supernatants were collected, filtered, and precipitated. After centrifugation, lentiviral pellets were resuspended in TexMACS medium (Miltenyi Biotec, Bergisch Gladbach, Germany) supplemented with 3% human AB serum (Gemini Bio, West Sacramento, CA) and aliquoted at -80°C until use.

Primary human T cells were isolated by magnetic bead separation using anti-CD4 and anti-CD8 microbeads (Miltenyi Biotec). On Day 0, T cells were activated using human T-Cell TransAct (40 μl per 1x10^6^ cells) and cultured in TexMACS medium with 3% human AB serum, 12.5 ng/mL human IL-7 (Miltenyi Biotec) and 12.5 ng/mL human IL-15 (Miltenyi Biotec). T cells were transduced with the respective lentiviral vectors on Day 1. Cells were expanded and supplemented with fresh media as appropriate and harvested on Day 10.

### 2.4 Tumor cell cytotoxicity and cytokine production by TRuC T cells

Stable luciferase-expressing tumor cells were plated in triplicates in a 96-well plate at 1x10^4^ cells per well in appropriate media for the tumor cell line used, and T cells were added at the indicated E:T ratios. After 24-hour culture, 50% of the culture supernatant was removed and preserved at -80°C for cytokine analysis. Tumor cell killing was determined using the Bright-Glo Luciferase Assay System (Promega, Madison, WI) according to the manufacturer’s protocol. Relative luminescence (RLU) was measured using the SpectraMax M5 plate reader (Molecular Devices, Sunnyvale, CA). The percentage of tumor lysis was calculated by the following formula:


$$\%\;\text{tumor cell lysis}=100\%\;\times \;\left(\frac{1-{\text{RLU}}^{\text{Tumor+T cell}}}{{\text{RLU}}^{\text{Tumor}}}\right)$$

Quantification of human cytokine levels was performed using a custom 4-plex Meso Scale Discovery (MSD) U-plex kit (Rockville, MD) according to the manufacturer’s instructions.

### 2.5 Evaluation of fratricide by CD70 TRuC T cells

Donor-matched T cells were thawed and activated using TransAct microbeads (40 µL/1x10^6^ cells, Miltenyi Biotec) overnight. After 18–24 hours, activated T cells were washed to remove the microbeads and non-activated and activated T cells were labeled with 5 µM CTV (Invitrogen, Eugene, OR) in phosphate-buffered saline (PBS) for 10 minutes at 37˚C. The reaction was quenched by addition of AB serum (Gemini Bio). CTV-labeled resting or activated non-transduced (NT) T cells were resuspended in complete media (RPMI with 10% FBS) and co-cultured with anti-CD70 TRuC T cells (normalized to %TRuC^+^) or additional unlabeled resting NT T cells at indicated E:T ratios. The co-cultures were incubated at 37˚C, and after 48 hours, samples were evaluated by flow cytometry for non-TRuC T-cell loss. Killing of NT T cells was calculated using the following equation:


$$\%\;\text{Killing}=\frac{\left(\%\;\text{Experimental Death}-\%\;\text{Background Death}\right)}{\left(\%\;\text{Max. Cell Death}-\%\;\text{Background Death}\right)}$$

### 2.6 Antibodies and flow cytometry

Human T cells were identified using the markers CD45 (HI30, BD Biosciences Cat# 563792, RRID: AB_2869519 or BioLegend Cat# 304014, RRID: AB_314402 or Cat# 304024, RRID: AB_493761), CD3 (OKT3, BioLegend Cat# 317322, RRID: AB_2561911; UCHT1, BioLegend Cat# 300460, RRID: AB_2564380), CD4 (RPA-T4, BioLegend Cat# 300526, RRID: AB_493743; SK3, BioLegend Cat# 344616, RRID: AB_2028483) and CD8 (SK1, BioLegend Cat# 344710, RRID: AB_2044010 or BioLegend Cat# 344714, RRID: AB_2044006 or BioLegend Cat# 344732, RRID: AB_2564624), and anti-CD70 TRuC^+^ cells were identified with anti-human IgG, F(ab’)_2_ (Jackson ImmunoResearch Labs Cat# 109-545-006, RRID: AB_2337832). Additional markers were evaluated as indicated. Surface marker antibodies included: CD56 (Biolegend Cat# 318306, RRID: AB_604101), CD45RA (HI100, BioLegend Cat# 304140, RRID: AB_2563816), CCR7 (G043H7, BioLegend Cat# 353204, RRID: AB_10913813 or BioLegend Cat# 353208, RRID: AB_11203894), CD70 (113-16, BioLegend Cat# 355124, RRID: AB_2820006), CD69 (FN50, BioLegend Cat# 310912, RRID: AB_314847), PD-1 (EH12.2H7, BioLegend Cat# 329932, RRID: AB_2562256), LAG-3 (11C3C65, BioLegend Cat# 369314, RRID: AB_2629797), TIGIT (A15153G, BioLegend Cat# 372718, RRID: AB_2632933), CD27 (M-T271, BioLegend Cat# 356420, RRID: AB_2562603) and IL15Rα (JM7A4, BioLegend Cat# 330210, RRID: AB_2561440). Intracellular marker antibodies were evaluated as indicated including TCF1/7 (C63D9, Cell Signaling Technology Cat# 6709, RRID: AB_2797631 or Cell Signaling Technology Cat# 9066, RRID: AB_2797696) and Mcl-1 (LVUBKM, Thermo Fisher Scientific Cat# 51-9047-42, RRID: AB_2811847). Ex vivo evaluations were performed using anti-mouse CD45 (30-F11, BioLegend Cat# 103132, RRID: AB_893340; I3/2.3, BioLegend Cat# 147716, RRID: AB_2750449).

Flow cytometric staining was performed as follows: Cell samples were aliquoted into 96-well plates, washed with PBS and stained with LIVE/DEAD Fixable Blue viability dye (Invitrogen) diluted 1:1000 in PBS to distinguish live cells. Cells were washed with staining buffer (PBS with 2% FBS) followed by incubation for 20 minutes with 1:100 Anti-Human IgG, F(ab’)_2_. Cells were washed again with staining buffer and resuspended in the remaining surface stain antibody cocktail. Flow cytometry was conducted on a BD LSRFortessa X-20 cytometer.

For the competition assay to detect CD70 on anti-CD70 TRuC T cells, C10 TRuC T cells were stained with increasing concentrations of phycoerythrin-labeled anti-15F6 hIgGFc and incubated at 4°C for 15 minutes. Cell samples were washed with staining buffer and fixed with 150 µL of FluoroFix (BioLegend) for 30 minutes. Fixative was washed out, and the samples were resuspended in staining buffer before performing flow cytometric analysis.

For intracellular staining, cells were fixed using the eBioscience Foxp3/Transcription Factor Staining Buffer Set (Invitrogen) according to the manufacturer’s instructions. Cells were then incubated with intracellular antibody cocktail for 30 minutes. Samples were washed and resuspended in staining buffer for flow cytometric analysis.

### 2.7 pSTAT5 detection

T cells were thawed and rested overnight at 37˚C in serum-free TexMACS media. After 18–24 hours, the cells were washed and plated at 1x10^5^ cells per well in a 96-well plate in either RPMI-1640 alone or supplemented with 10 ng/mL IL-15 or 100 nM ruxolitinib (Selleck Chemicals, Houston, TX). Cells were then directly fixed 1:1 with pre-warmed Cytofix Fixation Buffer (BD Biosciences) for 10 minutes at 37°C, washed with PBS with 2% FBS and permeabilized with pre-chilled Phosflow Buffer III (BD Biosciences) for 30 minutes at 4°C. pSTAT5 signaling was determined by staining with APC-labeled anti-human pSTAT5 (A17016B.Rec, BioLegend Cat# 936906, RRID: AB_2892500) for 30 minutes at room temperature.

### 2.8 Autonomous persistence assay

T cells were plated at 1x10^6^ cells per well in a 48-well plate in 1 mL of complete media (RPMI-1640 with 10% FBS) alone or supplemented with either 100 IU/mL IL-2 or 10 ng/mL IL-15 and incubated at 37˚C. After 4 days, half of the cell suspension was removed for cell cytometric analysis to determine cell expansion and phenotype, and fresh media, either alone or supplemented with 100 IU/mL IL-2 or 10 ng/mL IL-15, was added to the wells and incubated at 37°C. This process was repeated every 3–4 days until Day 15. For evaluation of cell numbers via flow cytometry, ~5000 counting beads (123count eBeads, Invitrogen) were added to each sample prior to flow cytometric analysis.

### 2.9 Antigen-dependent persistence assay

T cells were co-cultured with target tumor cell lines at a 5:1 E:T ratio, with 1x10^6^ T cells and 0.2x10^6^ target tumor cells, in a 48-well plate in complete media alone or supplemented with 100 nM each of dasatinib, ruxolitinib, or tofacitinib (Selleck Chemicals) and incubated at 37°C. After 4 days, half of the cell suspension was removed for cell cytometric analysis to determine cell expansion and phenotype, and fresh media – supplemented with 100nM each of dasatinib, ruxolitinib or tofacitinib when indicated – was added back to the wells and incubated at 37°C. This process was repeated every 3–4 days until Day 15. For the evaluation of cell numbers via flow cytometry, ~5000 counting beads (123count eBeads) were added to each sample prior to flow cytometric analysis.

### 2.10 Repeated stimulation assay

C10 TRuC T cells and NT cells were co-cultured with target tumor cells lines at 1:1 E:T ratio, with 3.5x10^4^ total or TRuC T cells and 3.5x10^4^ target tumor cells, in a 96-well plate in complete media and incubated at 37°C. The co-cultures were rechallenged every 4 days with 3.5x10^4^ fresh target tumor cells. Every 3–4 days until Day 15, the culture supernatants were harvested and stored at -80°C for later cytokine analysis by U-PLEX, and the co-cultures were harvested for flow cytometric analysis to determine T-cell expansion and phenotype. Separate conditions were set up for harvest at each collection timepoint. For the evaluation of cell numbers via flow cytometry, ~5000 counting beads (123count eBeads) were added to each sample prior to flow cytometric analysis.

Identical conditions were set-up in a 96-well white-walled plate to determine tumor cell lysis by assessing the luciferase activity from residual live tumor cells using the Bright-Glo Luciferase Assay System (Promega, Fitchburg, WI) according to the manufacturer’s instructions.

### 2.11 NK cell transactivation assay

Bystander natural killer (NK) cell activation was measured in co-culture assays were performed using donor-matched primary human peripheral blood mononuclear cells (PBMCs). On Day 0, cryopreserved TRuC T cells (ADP-520, C10, or NT) and donor-matched PBMCs were thawed, washed, and counted. T cells were labeled with CTV and co-cultured with unlabeled PBMCs at a 1:1 ratio (1×10^5^ cells each) in complete media (RPMI-1640 with 10% of heat-inactivated FBS). Co-cultures were plated in 96-well round-bottom plates and incubated at 37°C, 5% CO_2_ for 72 hours in the presence or absence of 786-O RCC target cells.

At endpoint, cells were stained for lineage (CD45, CD3, CD56) and activation (CD69) markers. Live/dead discrimination was performed using LIVE/DEAD Fixable Blue viability dye. NK cells were identified by flow cytometric analysis as CD3^−^CD56^+^CTV^−^, and CD69 expression was used as a surrogate marker for activation.

Recombinant human IL-15 (10 ng/mL; Miltenyi Biotec) was included as a positive control for IL-15–mediated NK cell activation. All conditions were run in technical triplicates, and ~5,000 123count eBeads were added to each well before acquisition for normalization of absolute cell counts to measure cell type fold-expansion by flow cytometric analysis.

### 2.12 Antitumor efficacy in mouse xenograft models

Animals were handled under an Institutional Animal Care & Use Committee (IACUC) protocol managed by Charles River Accelerator and Development Lab and following the guidelines set forward by the Guide for the Care and Use of Laboratory Animals. Animal numbers per cohort were determined by power analysis, using standard deviations from kinetic models, an 80% power probability, and a 30% tumor volume reduction as the effect threshold (30). This analysis recommended four mice per group, but all studies included at least five mice per group.

6–8-week old female NSG (NOD.Cg-Prkdc^scid^ Il2rg^tm1Wjl^/SzJ; Strain# 005557, RRID: IMSR_JAX:005557) and NSG-MHC I/II DKO (NOD.Cg-Prkdc^scid^ H2-K1^tm1Bpe^ H2-Ab1^em1Mvw^ H2-D1^tm1Bpe^ Il2rg^tm1Wjl^/SzJ; Strain# 025216, RRID: IMSR_JAX:025216) mice were acquired from Jackson Laboratory (Bar Harbor, ME), and all animal studies were approved by the Charles River Laboratories Animal Care and Use Committees. For the systemic MOLM-13 AML model, 5x10^4^ MOLM-13-Luc cells were infused intravenously into the tail vein of NSG mice. Four days post-tumor injection, mice were randomized into treatment groups and infused with 10^7^ C10 TRuC, 15F6 TRuC or NT T cells. For the 786-O model, 3x10^6^ 786-O-Luc cells were implanted subcutaneously into NSG or NSG-MHCI/II DKO mice. Eighteen days post-tumor implant, tumor sizes reached ~150–200mm^3^, and mice were infused intravenously with 0.5–3x10^6^ anti-CD70 TRuC or NT T cells. For the ACHN model, 2x10^6^ ACHN-Luc cells were implanted subcutaneously into NSG-MHCI/II DKO mice and allowed to engraft for ~12 days to reach a tumor size of 150–200mm^3^. Randomized mice were infused intravenously with 1x10^6^ TRuC T cells or NT T cells. Across treatment groups, TRuC T cells were normalized to %TRuC^+^ by the addition of NT T cells as necessary. Tumor burden was determined by caliper measurements (Tumor volume = (length × width^2^)/2) and/or IVIS bioluminescence imaging (PerkinElmer, Waltham, MA) twice per week. Euthanasia was performed by trained staff and accomplished using CO_2_ asphyxiation followed by cervical dislocation, in accordance with our IACUC protocol.

### 2.13 Ex vivo sample processing and evaluation of TRuC T cells

For the ex vivo evaluation of TRuC T cells, submandibular blood was collected into ethylenediaminetetraacetic acid tubes and mice were euthanized for tumor excision at indicated timepoints. Blood samples were subjected to red blood cell lysis using ACK lysis buffer (Gibco) and washed with PBS to remove clotted lysed cells. Subcutaneous tumors were enzymatically digested using the Tumor Dissociation Kit, human (Miltenyi Biotec) and filtered to obtain a single cell suspension. Single cell blood and tumor cell suspensions were aliquoted and analyzed by flow cytometry to evaluate TRuC T-cell infiltration.

### 2.14 Statistical analysis

GraphPad Prism (RRID: SCR_002798) was used for statistical analyses. *In vitro* experiments were performed with three technical replicates. Treatment groups were compared via one-way or two-way analysis of variance (ANOVA), followed by Dunnett’s multiple comparison test, as appropriate. Ex vivo experiments were performed with three biological replicates, and treatment groups were compared using one-way ANOVA with Dunnett’s multiple comparison test. For *in vivo* studies, treatment groups were compared using a two-way repeated-measures ANOVA, followed by the Bonferroni *post hoc* test. Statistical significance was systematically defined as P<0.05.

## 3 Results

### 3.1 CD70 binders stratify into fratricide-prone and fratricide-resistant clones based on phenotype

We executed three strategies to generate high-affinity anti-CD70 antibody fragments for assimilation into the TRuC T-cell platform, including evaluation of variable heavy domain of heavy chain [VHH] (Track 1), fully human and naïve single chain variable fragment [scFv] (Track 2), and humanized hybridoma-derived scFv (Track 3) libraries (**Supplementary Figure 1A**). These campaigns generated an enriched CD70 antibody fragment library comprising 112 unique heavy-chain complementarity-determining region 3 (HCDR3) clonotypes that exhibited an affinity range of <1–56 nM (K_D_) for CD70, competing with CD27 for CD70 binding. Of these, 25 antibody fragments were formatted into and screened in the TRuC T-cell platform via N-terminal fusion to full-length human CD3ε.

Healthy donor CD4^+^ and CD8^+^ T cells were transduced using lentiviral vectors that encoded the candidate anti-CD70 TRuCs. TRuC T cells generated by fusion with Track 1 or Track 3 anti-CD70 binders exhibited CD70-dependent fratricide, characterized by poor T cell expansion, high CD69 expression, and terminally differentiated memory phenotype, with Track 3 also showing poor *in vitro* effector functions (properties summarized in **Supplementary Figure 1A**). TRuCs constituting anti-CD70 fragments derived from Track 2 and formatted as N-terminal fusion of variable heavy chain to variable light chain (vHvL), or vice versa (vLvH), yielded several candidates with >10-fold expansion (**Supplementary Figure 1B**) and/or TRuC^+^ surface staining above the >50% cutoff (**Supplementary Figure 1C**). Half of Track 2 TRuC T cells exhibited a CD70^low^/CD69^hi^ expression pattern that portends fratricidal activity, while one TRuC T-cell product showed a CD70^hi^/CD69^hi^ expression pattern suggestive of target-independent auto-activation (**Supplementary Figure 1D**). While several Track 2 TRuC T cells exhibited markers of fratricide and auto-activation and were eliminated from downstream development, a subset remained CD69^low^ and maintained a memory phenotype dominated by naïve and central memory populations (**Supplementary Figure 1E**), as ascertained through expression levels of CD45RA and chemokine receptor 7 (CCR7). Together, these data identified several Track 2-derived TRuC T-cell candidates with favorable fratricide-resistant phenotypes for further testing.

The *in vitro* potency of CD70 TRuC T cells was assessed by measuring their tumor lytic activity and inflammatory IL-2 and interferon-gamma (IFN-γ) cytokine release response to CD70^+^ tumors. TRuC T cells were titrated by E:T against luciferase-expressing tumor cell lines, THP1, ACHN and 786-O, exhibiting a range of CD70 expression levels (**Supplementary Figures 1F–H**). While five TRuC T-cell products efficiently killed all three tumor cell lines, the Track 2 C10 vLvH TRuC T cells secreted markedly higher levels of both IFN-γ and IL-2 in response to tumor culture compared with all other candidates. Overall, the C10 vLvH TRuC T-cell product demonstrated a fratricide-resistant phenotype (favorable expansion, high TRuC expression, favorable memory phenotype distribution, low tonic activation) and high on-target effector function (**Supplementary Figure 1I**). The superiority of the C10 vLvH TRuC T-cell product, hereafter referred to as C10 TRuC T cells, led to its selection as a lead candidate for further characterization and development.

### 3.2 Identification of a fratricide-resistant CD70 TRuC T-cell product

**Figure 1:**
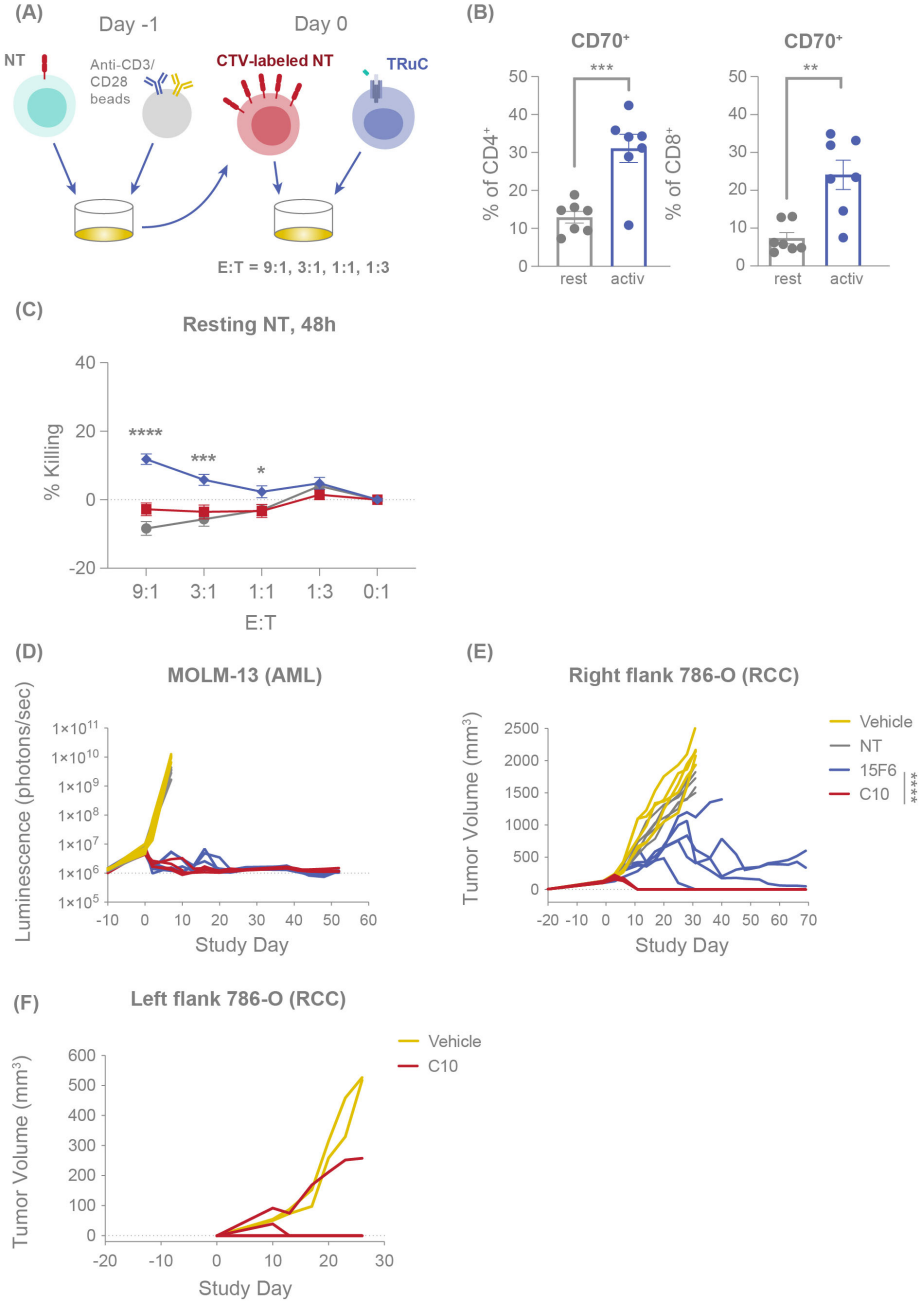
C10 TRuC T cells are fratricide resistant and exhibit potent and durable antitumor efficacy *in vivo*. **(A)** Experimental design of fratricide assay, in which CTV-labelled NT T cells are challenged with TRuC T cells at E:T of 9:1, 3:1, 1:1 and 1:3. **(B)** CD70 expression percentage was measured by flow cytometry for resting and anti-CD3/CD28 bead-activated CD4^+^ and CD8^+^ T cells. Columns depict the mean ± SEM. **(C)** Percentage of resting or activated CTV-labelled NT T cells killed when challenged with different E:T ratios of NT, C10 TRuC T cells and fratricidal 15F6 TRuC T cells for 48 hours, as evaluated by flow cytometry, mirroring the fold-change in CTV^+^ cells depicted in **Supplementary Figure 3A**. Each point represents the mean ± SEM. **(B, C)** report data from n=3 donors, all experiments performed in duplicate or triplicate, and data pooled. Quantification of the tumor burden in mouse models was performed following challenge with NT, 15F6 TRuC or C10 TRuC T cells. **(D)** The tumor burden of systemically administered MOLM-13-Luc (AML) cells following treatment was followed by means of bioluminescence. Changes in subcutaneous 786-O tumor volumes were measured using calipers, both following NT or TRuC T-cell treatment **(E)** and after re-challenging the opposing flank of the treated mouse with 786-O (RCC) cells **(F)**. *In vivo* data in **(D–F)** were obtained using T cells from a single donor and studies of 5 mice per group. *In vitro* treatment groups were compared via one-way ANOVA, followed by Dunnett’s multiple comparison test; *in vivo* treatment groups were compared using a two-way repeated-measures ANOVA, followed by the Bonferroni *post hoc* test; *p < 0.05, **p < 0.01, ***p < 0.001, ****p < 0.0001. AML, acute myeloid leukemia; ANOVA, analysis of the variance; CTV, CellTrace Violet; E:T, effector:target; NT, non-transduced; SEM, standard error of the mean; TRuC, T-cell receptor fusion construct.

We further investigated the ability of C10 TRuC T cells to avert bystander cytotoxicity by directly measuring their effector function response against CD70-expressing T cells compared with a fratricidal TRuC utilizing Track 1 anti-CD70 clone 15F6 (see experimental design, **Figure 1A**). As expected, activation of T cells with anti-CD3/anti-CD28 beads resulted in upregulated expression of CD70 in both CD4^+^ and CD8^+^ T cells (**Figure 1B**). Resting or activated T cells were labeled with CellTrace Violet (CTV), co-cultured with CD70-targeted TRuC T cells for 48 hours and evaluated for killing by flow cytometry. C10 TRuC T cells did not show significant killing of either resting or activated CD70-expressing CTV^+^ NT T cells (**Figure 1C**; **Supplementary Figure 2A**). In contrast, a TRuC product known to exhibit fratricide, clone 15F6, showed a low level of killing of resting T cells (~12%) and ~36% killing of activated T cells after 48 hours of co-culture (**Figure 1C**; **Supplementary Figure 2A**), comparable to the frequency of CD70^+^ expression present in the respective T cell populations (**Figure 1B**).

Studies of CD70 CAR T cells have previously indicated that a CD70-CAR cis interaction may occur on the CAR-T cell surface, masking the detection of CD70 and protecting the T-cell therapy from fratricide (29). Similarly, CD70 is not detected on C10 TRuC T cells (**Supplementary Figure 1D**). To evaluate whether a similar mechanism was occurring with C10 TRuC T cells, we performed a competition assay by titrating the addition of a fluorochrome-conjugated 15F6 binder, which shares an epitope bin with the C10 TRuC and therefore competes for CD70 binding on activated C10 TRuC T cells. Increasing concentrations of 15F6 resulted in the detection of CD70 on C10 TRuC T cells (**Supplementary Figure 2B**), suggesting that reduced detectability of CD70 on C10 TRuCs may be due to C10 TRuC-mediated cis masking of surface-expressed CD70, rather than CD70 depletion due to fratricide. Overall, these data confirm that C10 is a potent antitumor CD70 TRuC T-cell product that does not show significant fratricide or killing of normal bystander CD70^+^ T cells, at least partly due to the masking of surface CD70 by the TRuC itself.

### 3.3 Fratricide-resistant CD70 TRuC T cells exhibit potent and persistent antitumor efficacy *in vivo*

We next evaluated the *in vivo* antitumor efficacy of C10 compared with 15F6. In NOD SCID gamma (NSG) mice infused with the AML cell line MOLM-13-Luc, treatment with either C10 or 15F6 TRuC T cells effected a comparable and complete MOLM-13 tumor clearance that persisted to 50 days post-treatment (**Figure 1D**). Conversely, in a subcutaneous RCC (786-O) mouse model, C10 TRuC T cells induced potent and rapid tumor regression in all mice whereas 15F6 only delayed tumor growth and did not achieve full clearance in 4/5 mice (**Figure 1E**). The C10 TRuC T-cell-treated mice that showed complete RCC tumor regression were re-challenged with 786-O tumor cells on the opposite flank 43 days after treatment. Compared with naïve mice, which all exhibited 786-O tumor growth, only one of four re-challenged mice in the C10 TRuC T-cell-treated group showed tumor growth, with the prevailing 75% of other animals remaining tumor free (**Figure 1F**). This finding indicates that fratricide-resistant C10 TRuC T cells drive potent and persistent antitumor efficacy *in vivo*.

### 3.4 The addition of mbIL-15 to the CD70 TRuC T-cell product (ADP-520) induces a stem-like memory T-cell phenotype and retains potency

**Figure 2:**
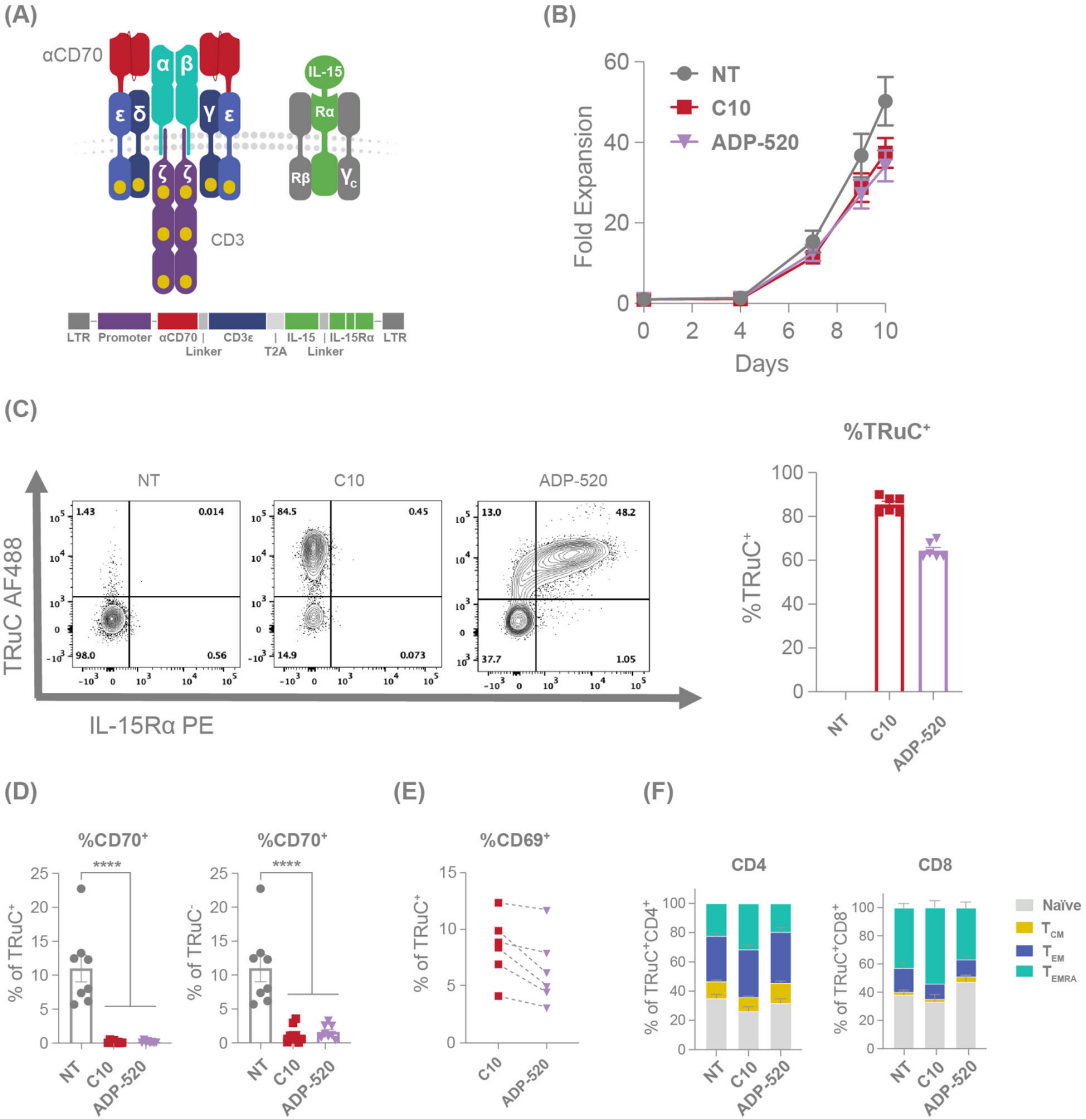
mbIL-15 enhanced TRuC T cells demonstrate an improved memory phenotype. **(A)** Illustration and schematic of the ADP-520 TRuC construct. **(B)** Comparative fold expansion of NT, C10 TRuC and ADP-520 TRuC T cells over 10 days of culture, with cell numbers determined using flow cytometry. **(C)** Illustrative flow cytometric analyses of TRuC and IL-15Rα expression in NT, C10 TRuC and ADP-520 TRuC T cells with a corresponding quantification of the percentage of TRuC^+^ cells within each type. Columns indicate the mean ± SEM of n=6 donor batches. **(D)** Comparative apparent CD70 expression on NT, C10 TRuC and ADP-520 TRuC T cells as a percentage of TRuC^+^ and TRuC^-^ cells by cytometric analysis. Columns indicate the mean ± SEM of eight replicate measurements. Treatment groups were compared via one-way ANOVA, followed by Dunnett’s multiple comparison test; ****p < 0.0001. **(E)** Cytometric quantification of CD69 expression in donor-matched TRuC^+^ C10 and ADP-520 TRuC T cells. **(F)** Phenotype proportion of TRuC^+^ NT, C10 TRuC and ADP-520 TRuC T cells by CD4^+^ and CD8^+^ subsets, reflecting CD45RA/CCR7 flow cytometric data (mean ± SEM): T_EMRA_ (terminal effector memory T cell), CD45RA^hi^/CCR7^low^; T_EM_ (effector memory T cell), CD45RA^low^/CCR7^low^; T_CM_ (central memory T cell), CD45RA^low^/CCR7^hi^; naïve, CD45RA^hi^/CCR7^hi^. AF, AlexaFluor; LTR, long terminal repeat; NT, non-transduced; PE, phycoerythrin; SEM, standard error of the mean; TRuC, T-cell receptor fusion construct.

Although C10 TRuC T cells demonstrated favorable characteristics relative to other screened candidates (**Supplementary Figure 1**) – including fratricide resistance and strong on-target effector function – several features were noted that might limit long-term persistence, including modestly elevated CD69 expression (**Supplementary Figure 1D**) and a trend towards increased effector memory cells re-expressing CD45RA (T_EMRA_) and reduced naïve cells (**Supplementary Figure 1E**). Utilizing IL-15 signaling to improve TRuC T-cell persistence, we generated ADP-520 TRuC T cells by combing the fratricide-resistant C10 TRuC receptor with membrane-bound IL-15:IL-15Rα fusion protein (mbIL-15) (**Figure 2A**). ADP-520 showed similar expansion during the TRuC T-cell product generation process (**Figure 2B**) and high transduction efficiency (~60–70% TRuC^+^) comparable to C10 TRuC T cells (80–90% TRuC^+^) (**Figure 2C**). ADP-520 showed masked CD70 expression that was comparable to C10 TRuC T cells (**Figure 2D**), and the addition of mbIL-15 did not increase target-independent auto-activation, as baseline levels of CD69 expression remained low and comparable to C10 TRuC T cells (**Figure 2E**). C10 TRuC T cells themselves showed a modest increase in terminally differentiated effector (T_EMRA_, CD45RA^+^CCR7^-^) cells (**Figure 2F**) and CD69 expression under monoculture conditions (**Supplementary Figure 1D**) compared to NT cells, possibly suggesting a low-level tonic C10 TRuC activation. However, these findings are not indicative of overt antigen-independent tonic signaling and instead demonstrate the low potential of C10 TRuC to elicit ligand-independent autoactivation. Comparatively, ADP-520 showed reduced T_EMRA_ CD4^+^ and CD8^+^ populations and an increased proportion of CD8^+^ naïve/stem cell memory (T_SCM_)-like (CD45RA^+^CCR7^+^) T-cell populations (**Figure 2F**). Since C10 and ADP-520 TRuCs were generated using identical media containing exogenous IL-15, these differences may under-estimate that impact of mbIL-15 co-expression on memory phenotype distribution and reflect the cis-signaling activity of mbIL-15, rather than differences in media formulation. ADP-520 also maintained the fratricide resistance of C10 TRuC T cells and exhibited little effect on the number of resting or activated CTV^+^ T cells (**Supplementary Figure 2C**).

**Figure 3:**
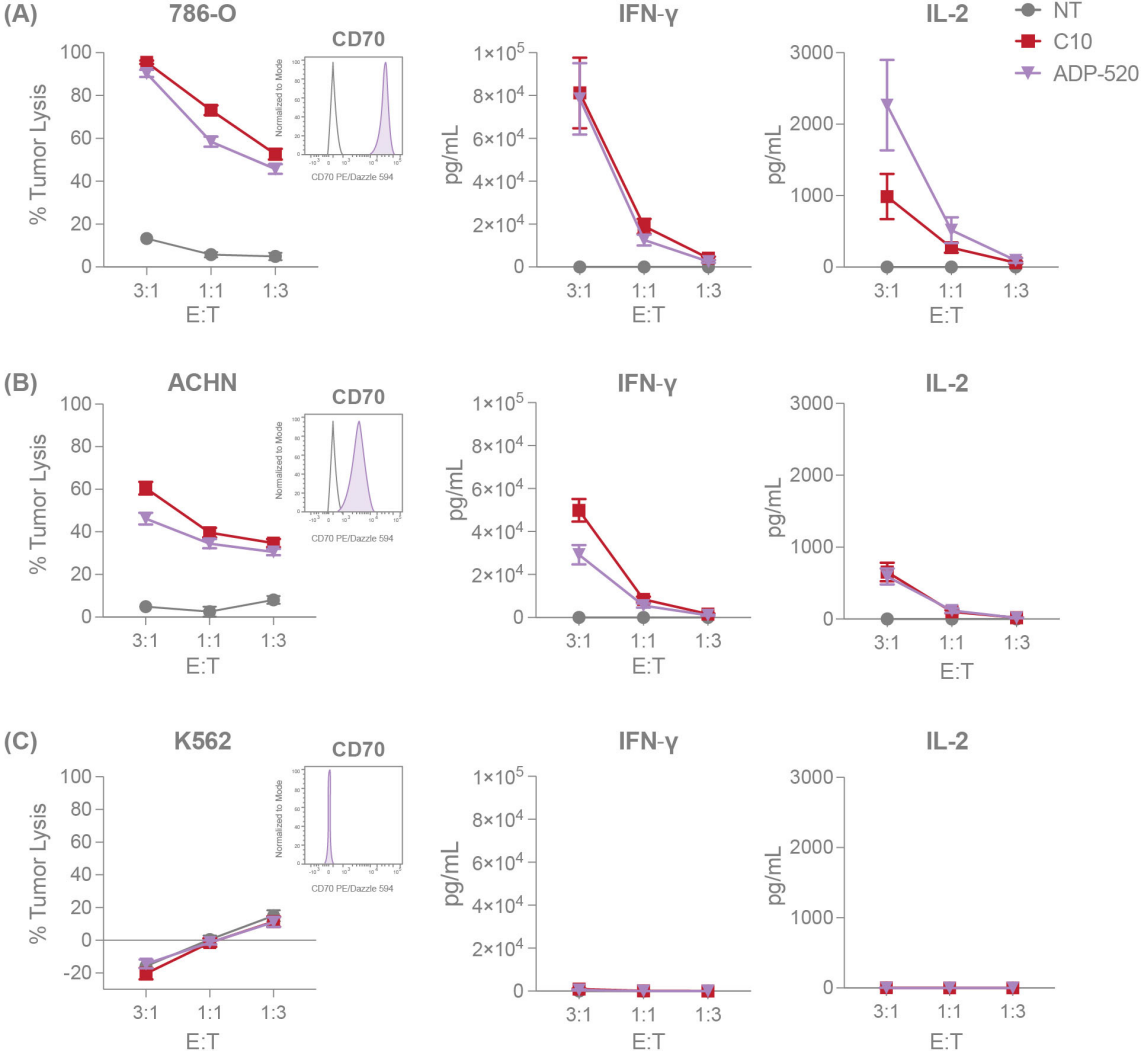
ADP-520 TRuC T cells demonstrate robust target-dependent potency *in vitro*. NT, C10 TRuC and ADP-520 TRuC T cells were titrated against tumor cell lines with different levels of CD70 expression. Percentages of tumor cell cytotoxicity, measured by luciferase assay, and IFN-γ and IL-2 cytokine secretion, determined by electrochemiluminescent detection, are shown for 786-O **(A)**, ACHN **(B)** and K562 **(C)** tumor cell lines at E:T ratios of 3:1, 1:1 and 1:3. Inset flow cytometry histograms demonstrate the CD70^hi^, CD70^mod^ and CD70^neg^ properties (shaded histograms) of the respective tumor cell lines compared to isotype controls (unshaded histograms). Data represent mean ± SEM for n=6 donor batches measured in triplicate. E:T, effector:target; PE, phycoerythrin; SEM, standard error of the mean; TRuC, T-cell receptor fusion construct.

Given that the mbIL-15 induces a reduced effector population and a higher proportion of naïve T cells within ADP-520 (**Figure 2F**), we next evaluated the potency of ADP-520 against CD70-expressing RCC cell lines, 786-O and ACHN. Compared with C10 TRuC T cells, ADP-520 displayed robust target-dependent cytokine production and antitumor activity against both the CD70^hi^ 786-O and CD70^mod^ ACHN cell lines, but not CD70^neg^ K562 (**Figures 3A–C**) or CD70-knockout (KO) 786-O cell lines (**Supplementary Figure 3A**). However, ADP-520 demonstrated higher IL-2 levels in response to CD70^hi^ 786-O (**Figure 3A**). Further, ADP-520 demonstrated robust cytotoxicity and cytokine production against additional CD70-expressing cell lines, including the MJ (**Supplementary Figure 3B**) and HuT78 (**Supplementary Figure 3C**) T-cell lymphoma cell lines, and the non-small cell lung cancer (NSCLC) line, NCI-H1975 (**Supplementary Figure 3D**). Collectively, these data show that ADP-520 TRuC T cells exhibit reduced signs of autoactivation and terminal differentiation while retaining strong efficacy against CD70-expressing target cells of multiple tumor indications.

**Figure 4:**
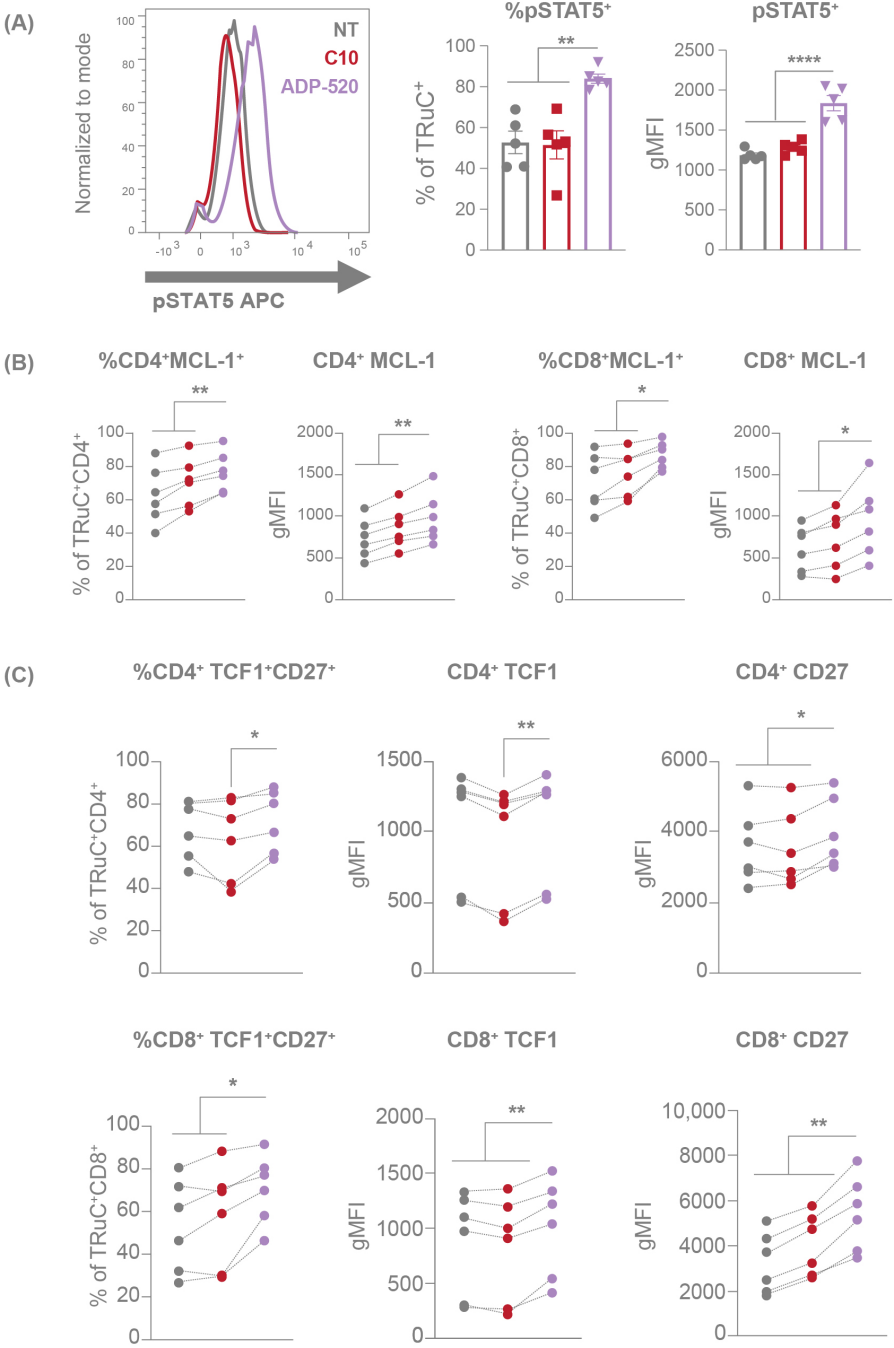
mbIL-15 enhancement activates the STAT5 pathway, resulting increased stemness of ADP-520 TRuC T cells. Markers of STAT5 activation in NT, C10 TRuC and ADP-520 TRuC T cells were quantified by flow cytometry. pSTAT5 expression is shown as a percentage of TRuC^+^ cells and gMFI of pSTAT5 staining **(A)**. Columns indicate the mean ± SEM. MCL-1 **(B)** and TCF1/CD27 **(C)** expression in donor matched CD4^+^ and CD8^+^ T cells is shown as a percentage of MCL-1^+^ or TCF1^+^/CD27^+^ TRuC^+^ cells and as gMFI of MCL-1, TCF1 and CD27 staining. Experiments were performed using n=6 donor batches and measured in triplicate. Treatment groups were compared via one-way ANOVA, followed by Dunnett’s multiple comparison test; *p < 0.05, **p < 0.01, ****p < 0.0001. APC, allophycocyanin; gMFI, geometric mean fluorescence intensity; NT, non-transduced; pSTAT5, phosphorylated signal transducer and activator of transcription 5; SEM, standard error of the mean; TRuC, T-cell receptor fusion construct.

IL-15 signals through the Janus kinase (JAK)1/3-signal transducer and activator of transcription (STAT) 5 pathway in T cells (17) and is known to promote the transcription of B-cell lymphoma 2 (BCL-2)-family genes, including anti-apoptotic induced myeloid leukemia cell differentiation protein 1 (*MCL1*) (31). To test whether the mbIL-15 enhancement engages IL-15 signaling pathways, we investigated the phosphorylation status of STAT5. TRuC T cells were rested overnight in serum and cytokine-free medium before harvesting for phospho-STAT5 (pSTAT5) detection. ADP-520 TRuC T cells showed increased resting pSTAT5 baseline signal compared with the C10 TRuC T cells and NT T cells (**Figure 4A**). In accordance, the ADP-520 TRuC T cells showed increased expression of MCL-1, particularly in CD8^+^ T cells (**Figure 4B**). Moreover, treatment of resting ADP-520 T cells with FDA-approved JAK1/2 inhibitor ruxolitinib reduced elevated STAT5 phosphorylation to levels comparable to untreated C10 TRuC T cells, suggesting that mbIL-15 confers higher basal IL-15 signaling to ADP-520 T cells (**Supplementary Figure 4A**). The reported ability of IL-15 to promote and maintain a T_SCM_ population capable of self-renewal (17) was supported by data that ADP-520 displays increased CD45RA^+^CCR7^+^ naïve T cells (**Figure 2F**). To further understand the impact of mbIL-15 on TRuC T-cell stemness, we evaluated the expression of the transcription factor, T cell factor 1 (TCF1, encoded by *TCF7*), an important regulator of T-cell development and differentiation that, in conjunction with CD27, marks quiescent T-cell populations that are capable of increased self-renewal (32, 33). ADP-520 TRuC T cells demonstrated an increased frequency of expression of both TCF1 and CD27 (**Figure 4C**) compared with C10 or NT T cells, indicating that stemness and self-renewal potential are increased by mbIL-15 in ADP-520 TRuC T cells.

### 3.5 ADP-520 demonstrates superior mbIL-15-driven autonomous and antigen-dependent persistence and expansion

To evaluate whether IL-15 expression in ADP-520 induces bystander NK cell expansion – a potential source of IL-15 associated toxicity – TRuC T cells were co-cultured with donor-matched PBMCs in the presence or absence of CD70^hi^ 786-O tumor cells (**Supplementary Figure 4B**). After 24 hours of co-culture, flow cytometry analysis revealed no increase in CD3^-^CD56^+^ NK cell frequency and negligible NK cell activation, as indicated by CD69 induction, in co-cultures with either ADP-520 or C10 TRuC T cells (**Supplementary Figure 4C**), unlike the robust NK cell activation observed upon treatment with recombinant IL-15 (positive control). These results indicate that mbIL-15 ADP-520 does not drive potent NK cell transactivation or proliferation, supporting its activity as a cis activating enhancement in ADP-520 with small impact on bystander NK cells and low risk of NK cell-associated toxicity.

**Figure 5:**
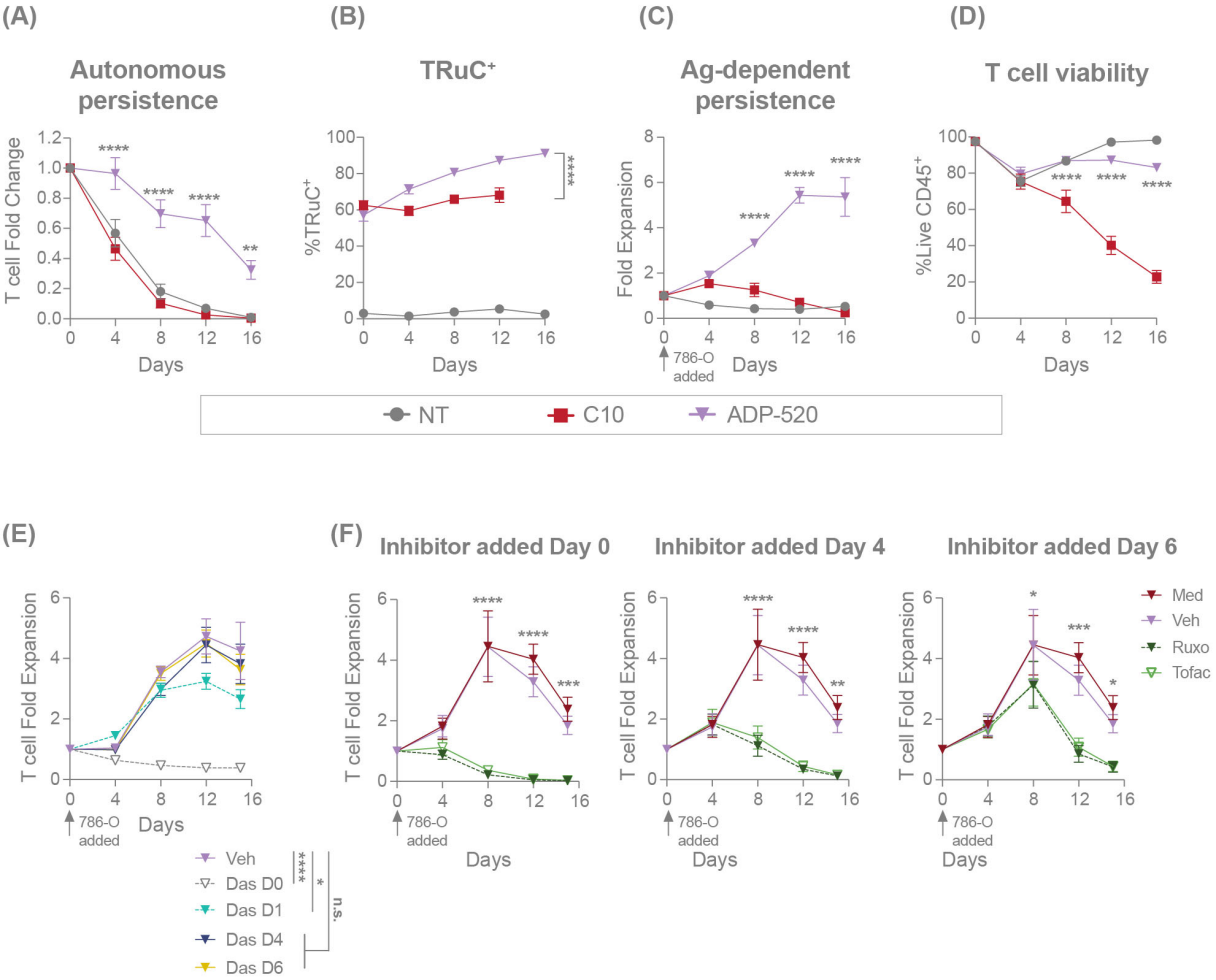
ADP-520 TRuC T cells demonstrate superior mbIL-15-dependent persistence in the presence or absence of exogenous cytokine or antigen stimulation. Flow cytometric analysis of the autonomic persistence of NT, C10 TRuC and ADP-520 TRuC T cells, showing growth curves in the absence of exogenous cytokines or antigen stimulation **(A)** and corresponding quantification of the percentage of TRuC^+^ cells at each timepoint **(B)**. Antigen-dependent persistence of NT, C10 and ADP-520 TRuC T cells, showing growth curves in the presence of 786-O tumor cells at a 5:1 E:T ratio **(C)** and corresponding quantification of the percentage of live CD45^+^ T cells at each timepoint **(D)**. Effect of TCR **(E)** or IL-15 **(F)** signaling inhibition on antigen-dependent expansion of ADP-520 TRuC T cells co-cultured with 786-O tumor cells at a 5:1 E:T ratio. The TCR signaling inhibitor dasatinib (Das), JAK1/2 inhibitor ruxolitinib (Ruxo) or JAK3 inhibitor tofacitinib (Tofac), or corresponding vehicle (Veh) or media (Med), were added to cocultures at the timepoints indicated and cell numbers determined by cytometric analysis. All data are mean ± SEM for n=3 donors, measured in triplicate. Treatment groups were compared via two-way ANOVA, followed by Dunnett’s multiple comparison test; *p < 0.05, **p < 0.01, ***p < 0.001, ****p < 0.0001. ANOVA, analysis of the variance; Das, dasatinib; E:T, effector:target; mbIL-15, membrane-bound interleukin-15; n.s., not significant; NT, non-transduced; SEM, standard error of the mean; TRuC, T-cell receptor fusion construct.

To further explore the impact of the mbIL-15 enhancement on TRuC T-cell persistence, we cultured TRuC T cells in the absence of antigen stimulation or exogenous cytokines. In contrast to C10 TRuC and NT T cells, which died rapidly in the absence of supportive cytokines, ADP-520 demonstrated enhanced autonomous persistence throughout 16 days of culture (**Figure 5A**). The TRuC^+^ population within ADP-520 was enriched from ~60% TRuC^+^ in the starting population to >80% by Day 16 of culture (**Figure 5B**), in contrast to the modest enrichment of C10 TRuC T cells, indicating that the mbIL-15 enhancement acts in *cis*, promoting TRuC^+^ persistence. While NT and C10 TRuC T cells expanded significantly in response to IL-2 or IL-15 cytokine stimulation, ADP-520 maintained consistent cell numbers throughout 16 days of culture (**Supplementary Figure 5A**). The ADP-520 TRuC^+^ population did not enrich in the presence of exogenous IL-2 and decreased to a small extent in response to IL-15 (**Supplementary Figure 5A**). Thus, ADP-520 TRuC T cells display increased cytokine-free persistence and exhibit diminished response to exogenous γ-chain cytokines acting through IL-2Rβ.

Next, we investigated the impact of mbIL-15 on antigen-dependent persistence. TRuC T cells were challenged with CD70^hi^ 786-O tumor cells at an E:T ratio of 5:1 and T-cell numbers were assessed over time. After single stimulation with CD70^hi^ 786-O tumor cells, C10 TRuC T cells showed initial expansion during tumor cell clearance at Day 4, followed by T-cell contraction to Day 16 of culture (**Figure 5C**). ADP-520 killed tumor cells and expanded to Day 12 before reaching stasis at Day 16 (**Figure 5C**). Accordingly, ADP-520 demonstrated drastically improved viability of T cells throughout the 16-day culture compared with C10 TRuC T cells (**Figure 5D**). Similar results were observed with CD70^mod^ ACHN tumor cells (**Supplementary Figure 5B**). These data indicate that mbIL-15 enhancement promotes ADP-520 TRuC T-cell persistence and antigen-dependent expansion.

We further explored the mechanism of action of the mbIL-15-mediated enhancement of T-cell expansion using pharmacological inhibitors. To confirm that initial ADP-520 expansion is regulated by primary antigen stimulation, we added the Src kinase and TCR signaling inhibitor dasatinib (34) to the ADP-520–786-O co-culture at various timepoints (**Figure 5E**). The addition of dasatinib at Day 0 or Day 1 after co-culture blocked initial T cell activation and reduced overall ADP-520 T-cell expansion in this assay. However, dasatinib added subsequent to tumor clearance (Day 4 or Day 6) had no impact on ADP-520 expansion (**Figure 5E**). To confirm that this long-term T-cell persistence is driven by mbIL-15, we targeted the IL-15 signaling pathway using JAK1/2 inhibitor ruxolitinib and JAK3 inhibitor tofacitinib. As expected, both inhibitors blocked ADP-520 T-cell expansion when added at Day 0, Day 4 or Day 6 (**Figure 5F**). These findings substantiate the role of mbIL-15 signaling in the long-term expansion of ADP-520 T cells following tumor clearance.

### 3.6 ADP-520 demonstrates improved persistence and resistance to exhaustion during chronic stimulation *in vitro*

**Figure 6:**
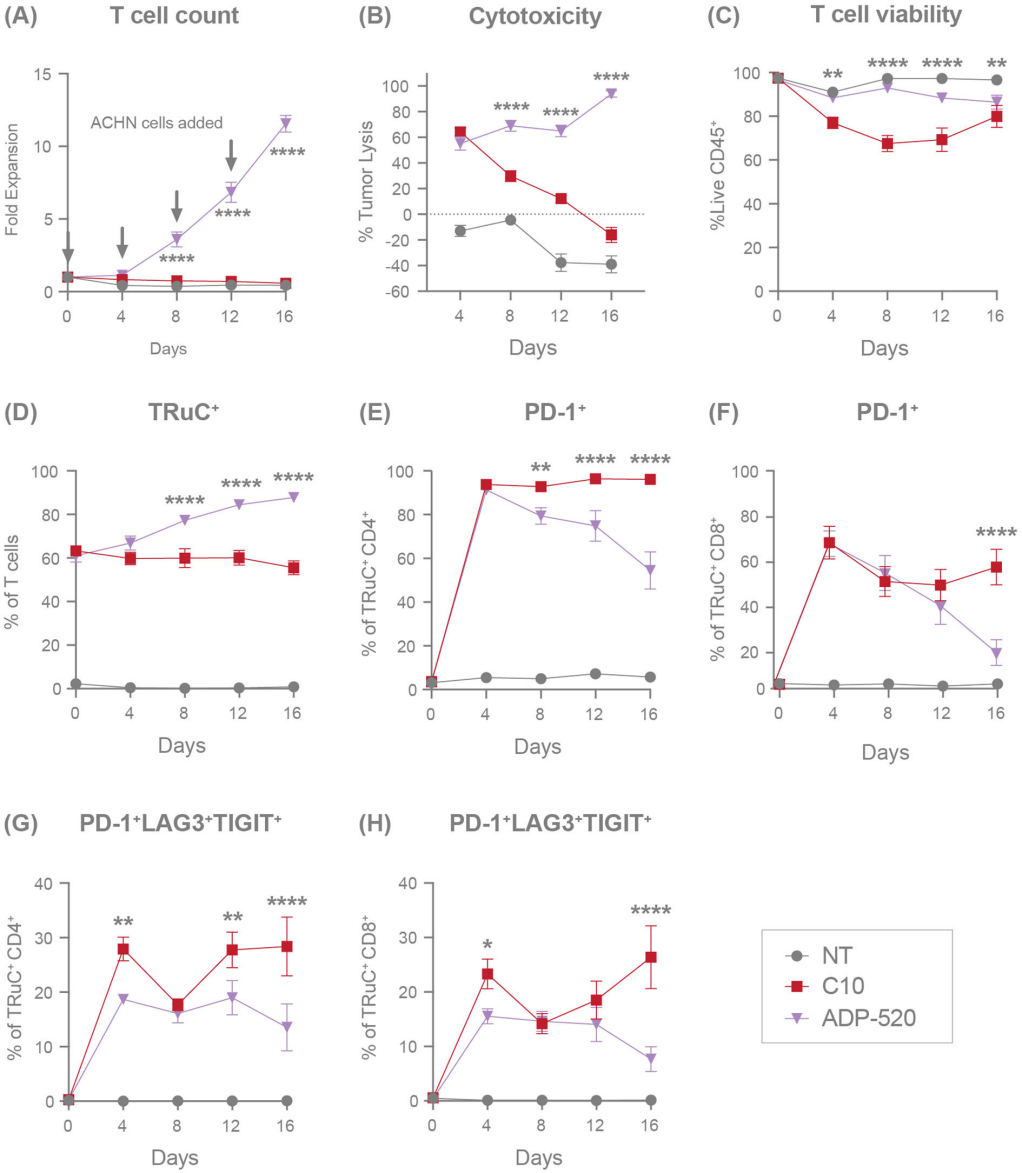
ADP-520 TRuC T cells demonstrate superior persistence, expansion, and resistance to exhaustion during chronic antigen stimulation *in vitro*. Flow cytometric characterization of response of NT, C10 TRuC and ADP-520 TRuC T cells to multiple rechallenges with ACHN tumor cells: fold expansion **(A)**, percentage of tumor cell lysis **(B)**, percentage of live CD45^+^ T cells **(C)**, and the percentage of TRuC^+^ T cells **(D)** are shown over 16 days of coculture. Corresponding expression of T-cell exhaustion markers are shown as a percentage of TRuC^+^ cells, either PD-1 alone **(E, F)** or the combination of PD-1, LAG3 and TIGIT **(G, H)**, for CD4^+^ and CD8^+^ populations, respectively. Arrows indicate timepoints of ACHN cell challenge. Data are mean ± SEM for n=3 donors, measured in triplicate. Treatment groups were compared via two-way ANOVA, followed by Dunnett’s multiple comparison test; *p < 0.05, **p < 0.01, ****p < 0.0001. ANOVA, analysis of the variance; NT, non-transduced; SEM, standard error of the mean; TRuC, T-cell receptor fusion construct.

We next sought to investigate the persistence of ADP-520 during conditions of repeated stress. ADP-520 TRuC T cells were cultured with CD70^mod^ ACHN tumor cells at a 1:1 E:T ratio and rechallenged with additional target cells every 4 days for a total of 16 days, with T-cell expansion and exhaustion evaluated by flow cytometry. While the C10 TRuC T cells never significantly expanded after initial or repeated challenge with ACHN, the ADP-520 TRuC T cells continued to expand throughout all three rechallenges (**Figure 6A**). Despite C10 and ADP-520 TRuC T cells showing similar tumor clearance (~60%) at Day 4, C10 failed to clear ACHN tumor cells while ADP-520 demonstrated durable and enhanced clearance of ACHN tumor during subsequent rechallenges (**Figure 6B**). ADP-520 also maintained significantly improved viability (~85-90%) throughout all rechallenges, whereas C10 TRuC T-cell viability was significantly decreased on Day 8 (**Figure 6C**). Only the ADP-520 TRuC T cells showed enrichment of TRuC^+^ T cells throughout all ACHN rechallenges (**Figure 6D**). Similar results were observed with CD70^high^ 786-O cells (**Supplementary Figure 5C**), as well MJ and HuT78 lymphoma lines (**Supplementary Figures 5D, E**). As expected, ADP-520 did not expand during re-challenge with CD70 KO target cell lines (**Supplementary Figure 5F**).

To further explore the mechanism of improved response and viability to repeated stress by ADP-520 TRuC T cells, we evaluated the expression of exhaustion markers on ADP-520 compared with C10 during repeated challenges with ACHN. Severe T-cell exhaustion is defined by the co-expression of several exhaustion markers, including PD-1, lymphocyte activation gene 3 (LAG-3) and T-cell immunoreceptor with Ig and ITIM domains (TIGIT). Both the C10 and ADP-520 TRuC T cells showed upregulated PD-1 expression equivalently at Day 4 in both CD4^+^ (**Figure 6E**) and CD8^+^ (**Figure 6F**) TRuC T cells. However, while C10 TRuC T cells maintained high PD-1 expression (~95% in CD4^+^, ~50% in CD8^+^) throughout the rechallenges, PD-1 expression was lower in ADP-520 by Days 8–16 (**Figures 6E, F**), an effect that was also observed in response to 786-O rechallenge (**Supplementary Figure 5G**). We found that ADP-520 showed a reduced frequency of PD-1^+^LAG3^+^TIGIT^+^ co-expressing CD4^+^ and CD8^+^ T cells at Day 4 after co-culture with ACHN (**Figures 6G, H**) and 786-O (**Supplementary Figure 5G**), suggesting that ADP-520 shows slower kinetics of exhaustion marker upregulation. This reduced PD-1^+^LAG3^+^TIGIT^+^ phenotype was maintained after subsequent re-challenges with ACHN tumor cells (**Figures 6G, H**). The distribution of T_CM_, T_Naïve_, T_EM_, and T_EMRA_ populations was also evaluated during tumor rechallenge, however both single and chronic antigen stimulation let to rapid and near-complete differentiation of both C10 and ADP-520 TRuC T cells toward the T_EM_ subset (data not shown). This convergence limited our ability to resolve meaningful differences in memory phenotype associated with the long-term persistence benefit of mbIL-15. Overall, ADP-520 demonstrates improved target-dependent potency, survival and resistance to exhaustion during repeated challenge with CD70-expressing target cell lines.

### 3.7 ADP-520 demonstrates enhanced tumor infiltration and antitumor efficacy *in vivo*

**Figure 7:**
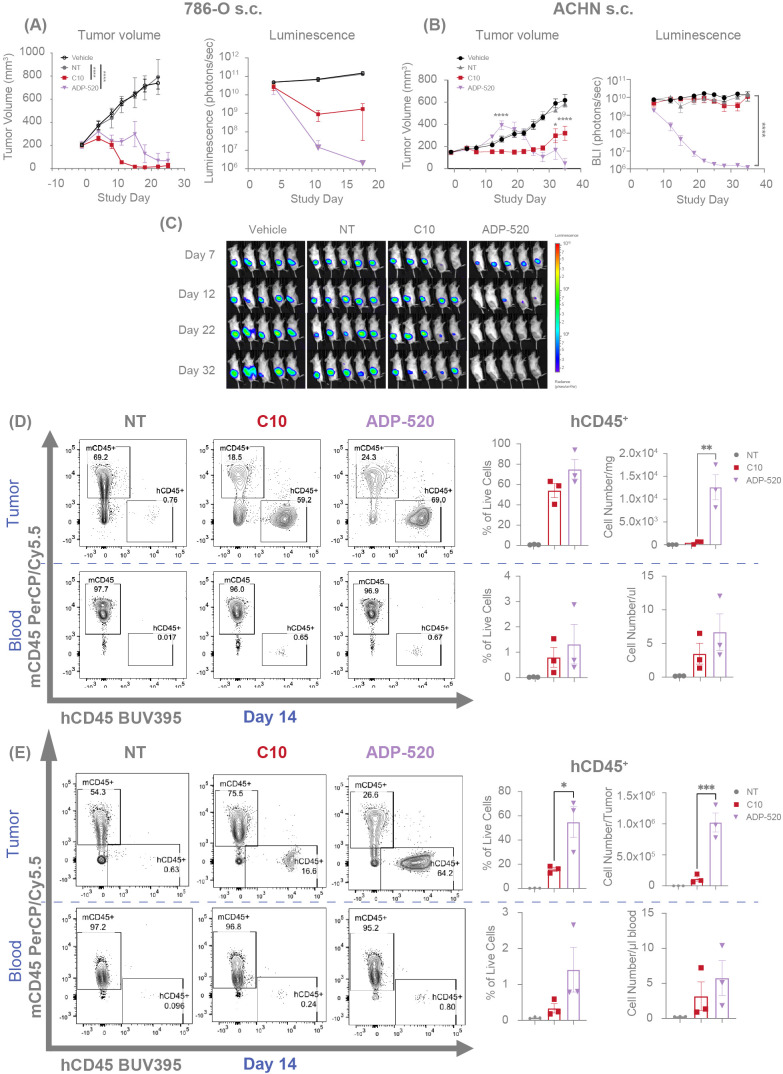
ADP-520 TRuC T cells exhibit enhanced intratumoral infiltration and anti-tumor efficacy *in vivo* with evidence of pseudoprogression. Temporal profile of tumor burden following treatment of mouse models of subcutaneous 786-O **(A)** and ACHN **(B)** tumors with intravenous NT, C10 TRuC or ADP-520 TRuC T cells. Bioluminescence images are depicted for the ACHN model **(C)**. Tumor burden is measured by means of caliper measurement of volume or bioluminescence intensity of luciferase-expressing tumor cells. *In vivo* data are mean ± SEM, n=1 donor per tumor and five mice per group. Treatment groups were compared using a two-way repeated-measures ANOVA, followed by the Bonferroni *post hoc* test. **(D, E)** TRuC T cell infiltration was analyzed by flow cytometric quantification of human vs mouse CD45 expression in the blood and tumor of the mice treated in **(A, B)**, respectively. Data presented as percentage of CD45^+^ live cells and number of cells per tumor or microliter of blood at 14 days post-treatment. Ex vivo data are mean ± SEM, n=3 mice per group. Treatment groups were compared using one-way ANOVA followed by Dunnett’s multiple comparison test; *p < 0.05, **p < 0.01, ***p < 0.001, ****p < 0.0001. ANOVA, analysis of the variance; BUV, Brilliant Ultra Violet; Cy, cyanine; NT, non-transduced; PerCP, peridinin-chlorophyll-protein; s.c., subcutaneous; SEM, standard error of the mean; TRuC, T-cell receptor fusion construct.

To evaluate the *in vivo* antitumor efficacy of ADP-520, we established human RCC tumor xenograft models for CD70^hi^ (786-O) or CD70^mod^ (ACHN) RCC tumors in NSG-major histocompatibility complex I/II (MHC I/II) double knock-out (DKO) mice. The C10 TRuC T cells demonstrated complete clearance of 786-O tumors (**Figure 7A**) and suppression of ACHN tumor growth (**Figure 7B**). Conversely, ADP-520-treated tumors exhibited apparent growth progression upon T-cell infusion, followed by tumor clearance in the 786-O (**Figure 7A**) and ACHN models (**Figure 7B**), when evaluated by tumor caliper measurements. Bioluminescence measurements of tumor status demonstrated that ADP-520 clearly and potently drove enhanced 786-O (**Figure 7A**) and ACHN (**Figures 7B, C**) tumor clearance, suggesting that early increases in tumor volume reflect tumor pseudoprogression, wherein tumor infiltration by immune cells presents as artificial and transient tumor enlargement (14, 35–37). To explore this further, tumors and blood were collected from treated mice and evaluated by flow cytometry. Although the frequency of human CD45^+^ (hCD45^+^) T cells was similar in the C10- and ADP-520-treated 786-O tumors, the ADP-520-treated tumors had ~25-fold increased total T-cell numbers compared with the C10-treated tumors (**Figure 7D**). Relatively few T cells were found in the blood of mice treated with either C10 or ADP-520 (**Figure 7D**). Similarly, the ADP-520-treated ACHN tumors contained a significantly increased frequency and number of hCD45^+^ T cells, with comparable numbers of T cells observed in the blood (**Figure 7E**). ADP-520 similarly enhanced antitumor efficacy and intratumoral T-cell infiltration in the CD70^low^ NSCLC tumor model NCI-H1975 (**Supplementary Figures 6A, B**). These data demonstrate that ADP-520 TRuC T cells provide potent *in vivo* efficacy against a range of CD70 tumor models enabled in part by enhanced tumor infiltration.

## 4 Discussion

In this study, we demonstrate that mbIL-15 enhanced CD70 TRuC is a potent, persistent and fratricide-resistant TRuC T-cell therapy with significant potential in CD70^+^ solid tumors. Physiologically, the appearance of CD70 is largely restricted to transient expression on antigen-activated T and B cells, natural killer cells and mature dendritic cells (23), but aberrant CD70 expression has been reported in many hematological cancers and several solid tumors (22, 26), including RCC where it is associated with metastasis and poor survival (23). CD70–CD27 signaling in the tumor microenvironment facilitates tumor immune escape by promoting immunosuppression, exhaustion and apoptosis in T cells (38–42). Accordingly, CD70 is an enticing target in immuno-oncology (22, 23, 26) and several targeted immunotherapies are currently in clinical development, including the monoclonal antibody cusatuzumab (43, 44), and the allogeneic CAR T-cell therapies CTX130 and ALLO-316, both of which are being investigated in RCC (45, 46).

The transient expression of CD70 on activated T cells means that CD70-targeted T-cell therapies are themselves at risk of self-killing, or fratricide, thus potentially limiting the viability and clinical efficacy of CD70-targeting ACTs (24, 45, 47). CTX130 circumvents this issue by knocking out the CD70 antigen in CAR-T cells using a CRISPR/Cas-9 editing system (27, 28). ALLO-316 was developed to be intrinsically fratricide-resistant, wherein the CD70-binding CAR is proposed to mask endogenous CD70 via a *cis* interaction (29). In the present study, candidate CD70 TRuC T cells were screened to eliminate fratricidal and autoactivating clones. The lead candidate, C10, exhibited desirable TRuC T-cell effector function, stem memory phenotype, *in vitro* tumor cytotoxicity, and *in vivo* efficacy against hematological and solid tumor xenografts. Critically, clone C10 also exhibits resistance to fratricide. Apparent loss of CD70 expression was observed among many of the screened CD70 binders, including C10; however, antibody staining titration of C10 TRuC T cells enabled detection of CD70 and C10 TRuC T cells did not exhibit tonic activation, suggesting that they exhibit a CD70 *cis*-masking mechanism reminiscent of ALLO-316 CAR T cells. Furthermore, C10 TRuC T cells exhibited negligible cytotoxicity against activated bystander T cells, suggesting that they possess a clinically advantageous aversion to suppressing host lymphocytes. The mechanistic basis favoring on-tumor cytotoxicity is unknown, but it may arise due to differences in the density of CD70 antigen expression, the nature of the targeted epitope recognized, or as a secondary consequence affiliated with apparent *cis*-masking of CD70. Finally, while CD70 knockout is a valid method to explore the role of endogenous CD70 in T-cell autoactivation, we did not pursue this approach. Given the robust persistence and durable antitumor activity of ADP-520 *in vitro* and *in vivo*, our focus was on developing a scalable, non-edited TRuC therapy that achieves fratricide resistance and functional persistence with minimal manufacturing complexity.

Persistence and sustained cytotoxic activity are key concerns in the development of effective ACTs, with various T-cell armoring and manufacturing approaches pursued to improve functional persistence within inhospitable tumor microenvironments (14). The inclusion of co-stimulatory elements has become a common approach in CAR-based therapies, with the aim of enhancing T-cell signaling and improving functionality. In a recent example, CD28 co-stimulation was found to significantly enhance the persistence and antitumor activity of allogeneic CD70-targeting CAR natural killer (NK) cells (48). While these promising results have prompted the initiation of a Phase 1/2 clinical trial in solid tumors, including RCC, challenges remain as NK infiltration into RCC tumors is reported to be limited compared with CD8 T cells (49) and yields a dysfunctional, poorly cytotoxic NK cell phenotype (50). Targeted disruption of key loci is an alternative approach to improved persistence. Allogeneic CTX130 CAR T cells avoid fratricide by CRISPR-mediated CD70 knockout and have demonstrated a promising objective response rate in patients with heavily pre-treated T-cell lymphoma during a Phase 1 clinical study. However, response rates to CTX130 were low in a phase 1 solid tumor RCC study, though one of 16 patients achieved a complete response, thereby demonstrating the promise of CD70-targeted T-cell therapies for RCC (45). In both instances, a relatively short persistence in peripheral circulation may adversely affect their duration of response (51). Accordingly, a next-generation version, CTX131, is currently under clinical investigation in RCC (52). CTX131 incorporates several gene edits, including disruption of Regnase-1, understood to promote a more functionally persistent phenotype (53). Indeed, higher frequencies of naïve, T_SCM_ and central memory (T_CM_) T-cell populations in T-cell therapies have been associated with improved clinical efficacy and anti-tumor potency compared with effector T-cell subsets (54–56); this is attributable to differences in persistence, expansion, self-renewal and differentiation potential (14, 57). IL-15 signaling has garnered attention for its association with supporting a T-cell T_SCM_ phenotype, self-renewal, metabolic fitness (17, 18, 58), and a pro-survival effect through the induction of anti-apoptotic MCL-1 (31). CAR T cells co-expressing soluble or membrane-bound IL-15 provide enhanced tumor cytotoxicity, increased expansion, decreased exhaustion and a higher frequency of T_SCM_ and T_CM_ cells compared with parental CAR T cells lacking IL-15 enhancement (18, 59, 60).

Clinical data from OBX-115, a tumor-infiltrating lymphocyte (TIL) product incorporating drug-inducible mbIL-15 (NCT05470283, NCT05086692), show modest NK cell expansion following IL-15 induction but do not report discontinuation of treatment due to toxicity (61). Notably, OBX-115 is administered at 150 x10^9^ TILs per patient, compared to the 50 x10^6^ million cell dose-level 1 typical of TRuC T cell clinical trials (12). Despite constitutive mbIL-15 expression, ADP-520 exhibited no evidence of uncontrolled proliferation, NK cell expansion, or toxicity in preclinical studies. These findings suggest that mbIL-15 can enhance persistence without provoking cytokine-related toxicity at doses relevant to ADP-520, though careful dose escalation and monitoring will remain important as clinical development advances.

In this study, we sought to improve the persistence and phenotype of C10 TRuC T cells by incorporating the mbIL-15 enhancement, yielding ADP-520 TRuC T cells. ADP-520 showed low basal activation while retaining selective on-target cytotoxicity against CD70^+^ tumors *in vitro* and avoiding on-target/off-tumor fratricide against activated T lymphocytes. Furthermore, relative to C10 TRuC T cells, ADP-520 was enriched in naïve and central memory populations and was characterized by increased IL-15-mediated signaling TRuC^+^ populations, as evidenced by increases in pSTAT5 and MCL-1 abundance. This increased mbIL-15 signaling was associated with improved autonomous persistence and TRuC^+^ T-cell enrichment in the absence of exogenous cytokines. Insensitivity to exogenous IL-15 or IL-2 by ADP-520 may suggest *cis* engagement of their shared IL-15Rβ/γ receptor subunits by surface-expressed IL-15Rα/IL-15. It is perhaps attributable to mechanistic signaling differences between soluble and endogenous or membrane-bound and trans-presented IL-15 (18), with endogenous or tethered IL-15 reportedly better supporting intratumoral and autonomous T-cell survival (18, 59). However, ADP-520 TRuC T cells did not demonstrate uncontrolled expansion, consistent with mbIL-15-functionalized CAR T cells (18). While inhibition of TCR signaling after tumor cell clearance did not impede long-term expansion of ADP-520 T cells, inhibition of mbIL-15 signaling using JAK inhibitors blocked expansion, demonstrating that incorporation of mbIL-15 supports autonomous and antigen-dependent persistence and expansion of ADP-520 TRuC T cells.

Exhaustion is a key impediment to the persistence and potency of T-cell immunotherapies and a substantial barrier to their successful application in solid tumors (62). Here, we show that ADP-520 TRuC T cells are superior to C10 TRuC T cells, exhibiting resistance to exhaustion during chronic antigen stimulation *in vitro*. Amidst repeated CD70^+^ tumor challenges, ADP-520 TRuC T cells showed slower kinetics and reduced co-expression of exhaustion markers, such as PD-1, the receptor for PD-L1, whose intratumoral expression was linked to poor clinical efficacy of gavo-cel TRuC T-cell therapy (12). Additionally, ADP-520 TRuC T cells maintained superior expansion, viability and cytotoxicity during antigen re-challenges.

Collectively, these features speak to an armored TRuC T-cell phenotype with the potential to maintain proliferative capacity and resist exhaustion in the context of chronic stimulation, which offers advantages in targeting solid tumors *in vivo*. Indeed, compared with C10, mbIL-15 engineered ADP-520 TRuC T cells demonstrated superior antitumor activity in CD70^+^ xenograft mouse models. ADP-520 TRuC T cells achieved potent tumor clearance in several solid tumor models, demonstrating superior clearance to C10 TRuC T cells, particularly in ACHN tumors characterized by moderate CD70 expression. Infiltration of solid tumor tissue remains an obstacle for ACTs even when armored against the immunosuppressive tumor microenvironment (1, 2, 4). RCC tumors may represent an ideal target in this regard, owing to their high expression of the CD70 target antigen (63) and permissiveness to T-cell infiltration (19). Our *in vivo* work suggests ADP-520 exhibits mild intratumoral pseudoprogression, transient post-treatment tumor site inflammation previously described for a number of immunotherapies, including ACTs, and reflecting the intratumoral accumulation of infiltrating immune cells (36). Accordingly, ADP-520 TRuC T-cell-treated tumors were marked by substantially increased numbers of infiltrating hCD45^+^ cells relative to mice receiving C10 TRuC T cells, demonstrating the tumor-penetrating potential of ADP-520 TRuC T cells. Such behavior was observed in the MSLN-targeted gavo-cel, where treatment enhanced CD3^+^CD8^+^ T-cell infiltration of the tumor and TRuC T cells were found to preferentially expand and persist in MSLN^+^ tumor tissue long after they became undetectable in peripheral blood (12).

A key limitation of this study is the reliance on xenograft tumor models to predict potential efficacy in humans. As preclinical models such as this do not provide insight into the interactions between TRuC-T cells and the host immune system and do not fully recapitulate the immunosuppressive tumor microenvironment of human solid tumors, they may overestimate the effector function of ADP-520 (64, 65). Additionally, while this study reports the IFN-γ and IL-2 production of activated ADP-520, the broader effector molecule profile, especially under conditions of prolonged antigen stimulation (66). Finally, while ADP-520 showed limited upregulation of exhaustion markers PD-1, LAG-3 and TIGIT during chronic antigen exposure, epigenetic and transcriptional profiling may provide a deeper characterization of an apparent exhaustion-resistant stem-like memory phenotype. In particular, observations of the dynamics of exhaustion-associated transcription factors, such as TOX (thymocyte selection-associated high mobility group box), NR4A (nuclear receptor subfamily 4A), BATF (basic leucine zipper transcription factor, ATF-like) and TCF1, may provide further evidence for the plasticity of ADP-520 cells (67).

In conclusion, we have generated promising preclinical evidence supporting the use of ADP-520 armored TRuC T cells in treating CD70^+^ tumors. This fratricide-resistant T-cell immunotherapy demonstrates enhanced expansion, exhaustion resistance and persistence afforded by mbIL15-mediated signaling, with potent target-specific antitumor capabilities demonstrated across several cell lines *in vitro* and *in vivo*. We aim to advance ADP-520 to clinical study with an eye towards developing a first-in-class autologous and armored CD70-targeted cell immunotherapy for addressing the unmet needs of both hematologic malignancies and solid tumors, such as RCC.

## Data Availability

The raw data supporting the conclusions of this article will be made available by the authors, without undue reservation.

## Supplementary material

Supplementary Figure 1 Identification of a potent CD70-targeted TRuC T cell. **(A)** Summary of anti-CD70 antibody fragment screening strategies. **(B)** Fold expansion of Track 2 TRuC T cells after 10 days of culture, with cell numbers determined using flow cytometry, shown as mean fold expansion of n=3 donors, each a single reading. **(C–E)** Representative flow cytometric analyses (and corresponding quantifications) of TRuC T cells generated from different Track 2 anti-CD70 fragments, showing the proportion of TRuC+/CD3+ cells, and expression levels of CD70/CD69 and CD45RA/CCR7 in TRuC+ cells, respectively. The percentage of cells in the indicated gates is shown. CD45RA/CCR7 expression is quantified in terms of corresponding T cell phenotype: T_EMRA_ (terminal effector memory T cell), CD45RA_hi_ /CCR7_low_; TEM (effector memory T cell), CD45RA_low_/CCR7_low_; TCM (central memory T cell), CD45RA_low_/CCR7_hi_; naive, CD45RA_hi_/CCR7_hi_. **(F –H)**Titration of Track 2 TRuC T cells against CD70+ tumor cell lines; shown are flow cytometric quantification of percentage of CD70+ cells in THP1, ACHN and 786-O cell lines, respectively. Percentages of tumor cell cytotoxicity, measured by luciferase assay, and IFN-γ and IL-2 cytokine secretion, determined by electrochemiluminescent detection, are shown for each corresponding tumor cell line at TRuC T cell:tumor cell (E:T) ratios of 3:1, 1:1 and 1:3. Columns indicate the mean ± SEM of n=3 donors. **(I)** Heat map depicting the relative exhibition of key beneficial TRuC Tcell properties amongst the Track 2 TRuC T-cell candidates, normalized to value of NT. Ab, antibody; AF, AlexaFluor; APC, allophycocyanin; BV, Brilliant Violet; Cy, cyanine; Fc, fragment crystallizable region; NT, non-transduced; PE, phycoerythrin; scFV, single-chain variable fragment; SSC-A, side-scattering area; TRuC, T-cell receptor fusion construct; VHH, variable domain of heavy chain.

Supplementary Figure 2 Lack of CD70 expression on C10 TRuC T cells is due to masking not T-cell fratricide. **(A)** Fold change in the number of resting or activated CTV+ NT cells when challenged with NT, C10 TRuC or 15F6 TRuC T cells for 48 hours. **(B)** Representative flow cytometric analyses of CD70 and CD27 expression on C10 TRuC T cells from a single donor, showing increased unmasking of CD70 upon titration with CD70-binding 15F6 antibody fragment. Clockwise from the top-left quadrant, the percentage of cells in CD70hi/CD27low, CD70hi/CD27hi, CD70low/CD27hi and CD70low/CD27low gates are shown. **(C)** Change in numbers of resting and activated CTV-labelled NT cells when challenged with different E:T ratios of NT, C10 TRuC or ADP-520 TRuC T cells for 48 hours, as evaluated by flow cytometry. Data depicted in terms of percentage CTV+ NT cells killed and fold-change in CTV+ cells. (A) and (C) show the mean ± SEM for n=2 donor T cell batches measured in technical triplicate. BV, Brilliant Violet; CTV, CellTrace Violet; E:T, effector:target; NT, non-transduced; PE, phycoerythrin; SEM, standard error of the mean; TRuC, T-cell receptor fusion construct.

Supplementary Figure 3 ADP-520 TRuC T cells demonstrate in vitro efficacy against multiple CD70-expressing tumor cell lines. Titration of NT, C10 TRuC and ADP-520 TRuC T cells against tumor cell lines with different levels of CD70 expression, as demonstrated by flow cytometry histograms (shaded histogram = CD70, dashed histogram = CD70 KO, unshaded histogram = negative control). Percentages of tumor cell cytotoxicity, measured by luciferase assay, and IFN-γ and IL-2 cytokine secretion, determined by electrochemiluminescent detection, are shown for 786-O CD70 KO**(A)**, CD70hi MJ**(B)**, CD70mod HuT78 **(C)** and CD70lo NCI-H1975 **(D)** tumor cell lines at E:T ratios of 3:1, 1:1 and 1:3. Cytotoxicity/effector function is compared between wild-type and CD70 KO 786-O lines (A). Data represent n=2 donor batches measured in technical triplicate. E:T, effector:target; KO, knockout; PE, phycoerythrin; TRuC, T-cell receptor fusion construct.

Supplementary Figure 4 ADP-520 TRuC T-cells are non-responsive to exogenous IL-15 stimulation and constitutive mbIL-15 expression induced negligible NK cell activation. **(A)** Flow cytometric analysis of pSTAT5 expression in NT, C10 TRuC and ADP-520 TRuC T cells in response to exogenous IL-15 stimulation or JAK1/2 inhibition. Histograms depict representative pSTAT5 expression in TRuC+ and TRuC- populations from a single donor, with column chart showing mean (± SEM) pSTAT5 gMFI quantification in the TRuC+ population of n=6 donors measured in technical triplicate. **(B)** Flow cytometric quantification bystander NK cell expansion, measured by fold-expansion at 72-hour of co-culture, relative to Hour 0, in the presence or absence of CD70^+^ 786-O tumor cells during 1:1 co-culture with mock treatment (no T cells), donor-matched NT T cells, C10 TRuC T cells, or ADP-520 T cells. Recombinant human IL-15 (10 ng/mL final concentration) was used as positive control to induce NK activation. **(C)** Frequency of CD69 positive CD3-/CD56^+^ NK cells measured at 24 hours after co-culture treatment. Recombinant human IL-15 (10 ng/mL final concentration) was used as positive control to induce NK activation. Data are plotted as mean ± SEM from experiments performed using at least two donor T cell batches with measurements performed in triplicate. CD, cluster of differentiation; gMFI, geometric mean fluorescence intensity; IL, interleukin; JAK, Janus kinase; mbIL-15, membrane-bound IL-15; NK, natural killer; NT, non-transduced; PBMC, peripheral blood mononuclear cell; pSTAT5, phosphorylated signal transducer and activator of transcription 5; SEM, standard error of the mean; TRuC, T-cell receptor fusion construct.

Supplementary Figure 5 ADP-520 cells show improved T-cell expansion, persistence and resistance to exhaustion. **(A)** Flow cytometric quantification of NT, C10 TRuC and ADP-520 TRuC T-cell expansion and percentage of TRuC+ cells in the presence of exogenous cytokine stimulation (IL-15 or IL-2). **(B)** Antigen-dependent persistence of NT, C10 TRuC and ADP-520 TRuC T cells, showing growth curves in the presence of ACHN tumor cells at a 5:1 E:T ratio and corresponding quantification of the percentage of live CD45+ T cells at each timepoint. Expansion and cytotoxicity are shown for NT, C10 TRuC and ADP-520 TRuC T cells in response to multiple challenges from 786-O **(C)**, MJ **(D)**, HuT78 **(E)** and 786-O CD70 KO tumor cells **(F)**. Arrows indicate timepoint of additional tumor cell additions to coculture. Corresponding T-cell viability (as percentage of live CD45+ cells) and percentage of TRuC+ cells also shown for the 786-O coculture. **(G)** Flow cytometric quantification of T-cell exhaustion markers (PD-1 or the combination of PD-1, LAG3 and TIGIT) as a percentage of CD4+ or CD8+ NT, C10 and ADP-520 TRuC+ cells during the multiple 786-O rechallenges depicted in Supplementary Figure 5C. Data are mean ± SEM of n=3 donors, measured in technical triplicate. Treatment groups were compared via two-way ANOVA, followed by Dunnett’s multiple comparison test; **p* < 0.05, ***p* < 0.01, ****p* < 0.001, *****p* < 0.0001. APC, allophycocyanin; KO, knockout; NT, non-transduced; pSTAT5, phosphorylated signal transducer and activator of transcription 5; SEM, standard error of the mean; TRuC, T-cell receptor fusion construct.

Supplementary Figure 6 In vivo efficacy of ADP-520 TRuC T cells in an NSCLC NCI-H1975 tumor model. **(A)** Tumor volume in a subcutaneous NSG MHC class I/II KO mouse xenografted with 5 x 10_6_ tumor cells/mouse and then treated after 15 days by intravenous infusion of 1.4 x 10^6^ NT T cells, or 1 x 10^6^ C10 TRuC or ADP-520 TRuC T cells. **(B)** Flow cytometric quantification of human vs mouse CD45 expression in the blood and tumor of the mice treated in Supplementary Figure 6A. Data presented as percentage of CD45+ live cells and number of cells per tumor or microliter of blood at 14 days post-treatment. Experiments were performed using T cells from a single representative human donor. In vivo data are mean ± SEM of n=5 mice per group, with treatment groups compared using a two-way repeated measures ANOVA, followed by the Bonferroni post hoc test. Ex vivo are mean ± SEM of n=3 mice per group, with treatment groups compared using one-way ANOVA followed by Dunnett’s multiple comparison test; **p* < 0.05, ***p* < 0.01, *****p* < 0.0001. ANOVA, analysis of the variance; KO, knockout; MHC, major histocompatibility complex; NSG, NOD SCID gamma; NT, non-transduced; SEM, standard error of the mean; TRuC, T-cell receptor fusion construct.
