# Supplementary figures and images for: Membrane-bound IL-15 co-expression powers a potent and persistent CD70-targeted TRuC T-cell therapy
Source: Front Immunol. 2025 May 30;16:1609658. doi: 10.3389/fimmu.2025.1609658 (PMC12162932; doi:10.3389/fimmu.2025.1609658)

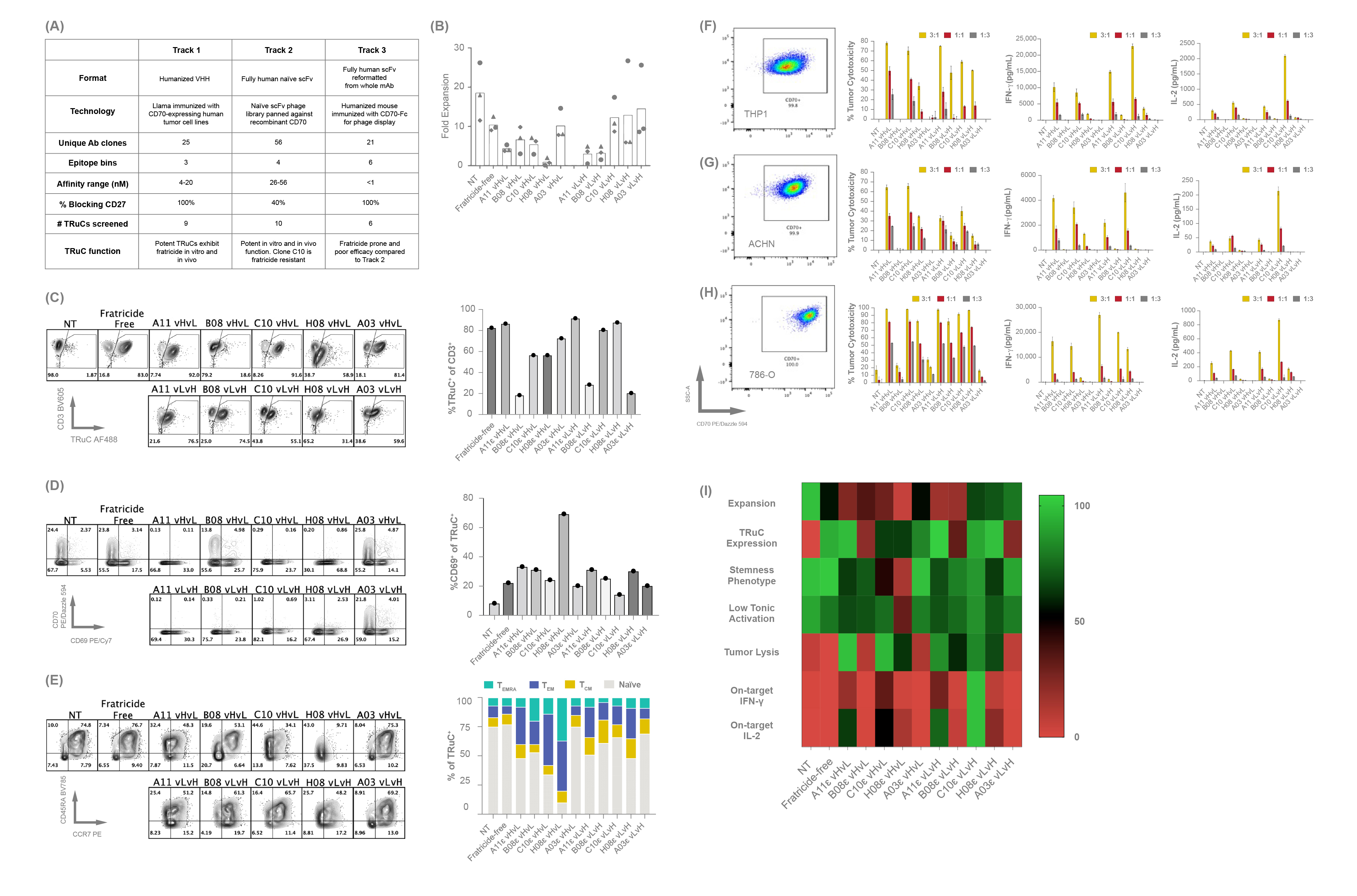

Supplement: Supplementary Figure 1 — Identification of a potent CD70-targeted TRuC T cell. (A) Summary of anti-CD70 antibody fragment screening strategies. (B) Fold expansion of Track 2 TRuC T cells after 10 days of culture, with cell numbers determined using flow cytometry, shown as mean fold expansion of n=3 donors, each a single reading. (C–E) Representative flow cytometric analyses (and corresponding quantifications) of TRuC T cells generated from different Track 2 anti-CD70 fragments, showing the proportion of TRuC+/CD3+ cells, and expression levels of CD70/CD69 and CD45RA/CCR7 in TRuC+ cells, respectively. The percentage of cells in the indicated gates is shown. CD45RA/CCR7 expression is quantified in terms of corresponding T cell phenotype: TEMRA (terminal effector memory T cell), CD45RAhi /CCR7low; TEM (effector memory T cell), CD45RAlow/CCR7low; TCM (central memory T cell), CD45RAlow/CCR7hi; naive, CD45RAhi/CCR7hi. (F –H)Titration of Track 2 TRuC T cells against CD70+ tumor cell lines; shown are flow cytometric quantification of percentage of CD70+ cells in THP1, ACHN and 786-O cell lines, respectively. Percentages of tumor cell cytotoxicity, measured by luciferase assay, and IFN-γ and IL-2 cytokine secretion, determined by electrochemiluminescent detection, are shown for each corresponding tumor cell line at TRuC T cell:tumor cell (E:T) ratios of 3:1, 1:1 and 1:3. Columns indicate the mean ± SEM of n=3 donors. (I) Heat map depicting the relative exhibition of key beneficial TRuC Tcell properties amongst the Track 2 TRuC T-cell candidates, normalized to value of NT. Ab, antibody; AF, AlexaFluor; APC, allophycocyanin; BV, Brilliant Violet; Cy, cyanine; Fc, fragment crystallizable region; NT, non-transduced; PE, phycoerythrin; scFV, single-chain variable fragment; SSC-A, side-scattering area; TRuC, T-cell receptor fusion construct; VHH, variable domain of heavy chain. [file Image1.jpg]

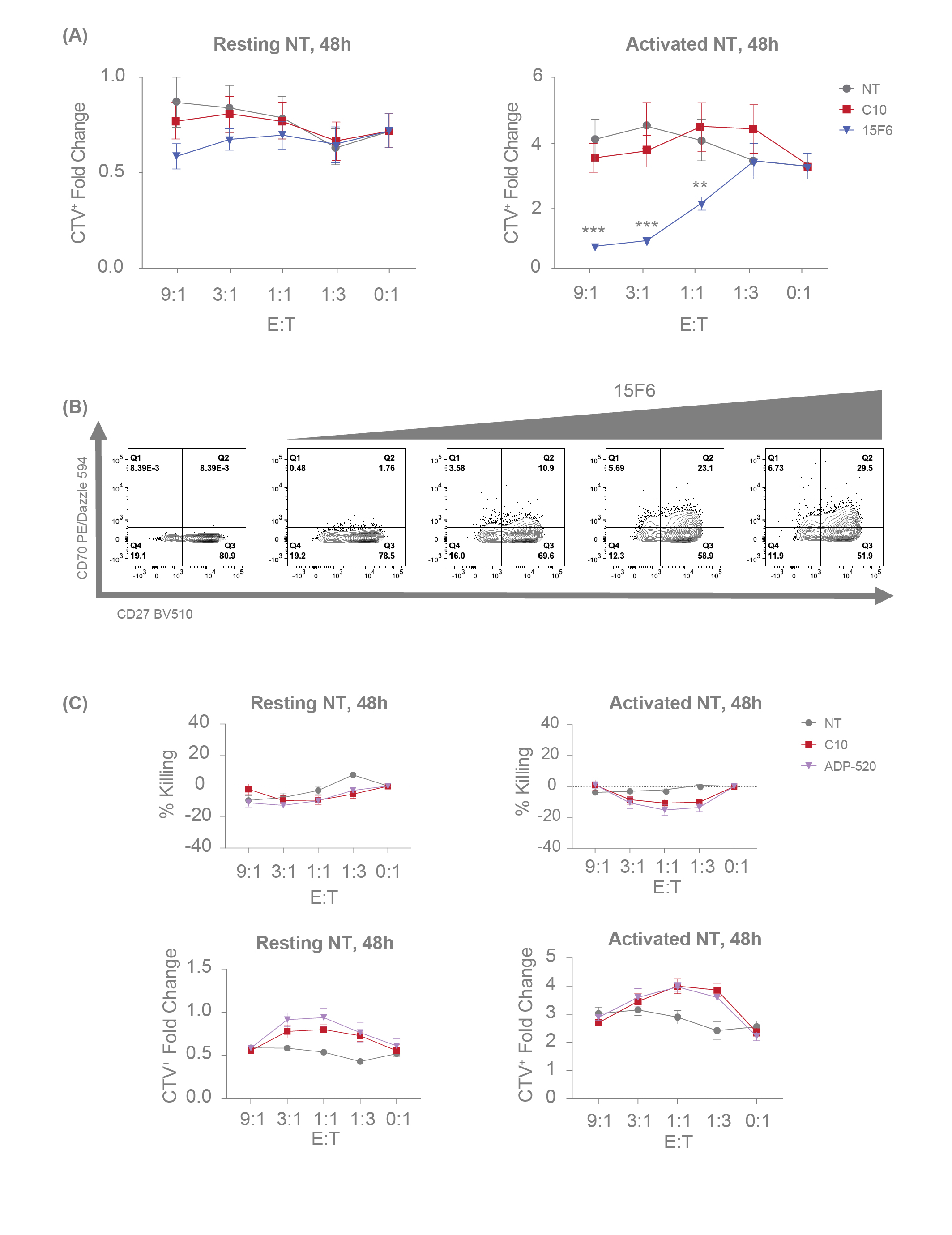

Supplement: Supplementary Figure 2 — Lack of CD70 expression on C10 TRuC T cells is due to masking not T-cell fratricide. (A) Fold change in the number of resting or activated CTV+ NT cells when challenged with NT, C10 TRuC or 15F6 TRuC T cells for 48 hours. (B) Representative flow cytometric analyses of CD70 and CD27 expression on C10 TRuC T cells from a single donor, showing increased unmasking of CD70 upon titration with CD70-binding 15F6 antibody fragment. Clockwise from the top-left quadrant, the percentage of cells in CD70hi/CD27low, CD70hi/CD27hi, CD70low/CD27hi and CD70low/CD27low gates are shown. (C) Change in numbers of resting and activated CTV-labelled NT cells when challenged with different E:T ratios of NT, C10 TRuC or ADP-520 TRuC T cells for 48 hours, as evaluated by flow cytometry. Data depicted in terms of percentage CTV+ NT cells killed and fold-change in CTV+ cells. (A) and (C) show the mean ± SEM for n=2 donor T cell batches measured in technical triplicate. BV, Brilliant Violet; CTV, CellTrace Violet; E:T, effector:target; NT, non-transduced; PE, phycoerythrin; SEM, standard error of the mean; TRuC, T-cell receptor fusion construct. [file Image2.jpg]

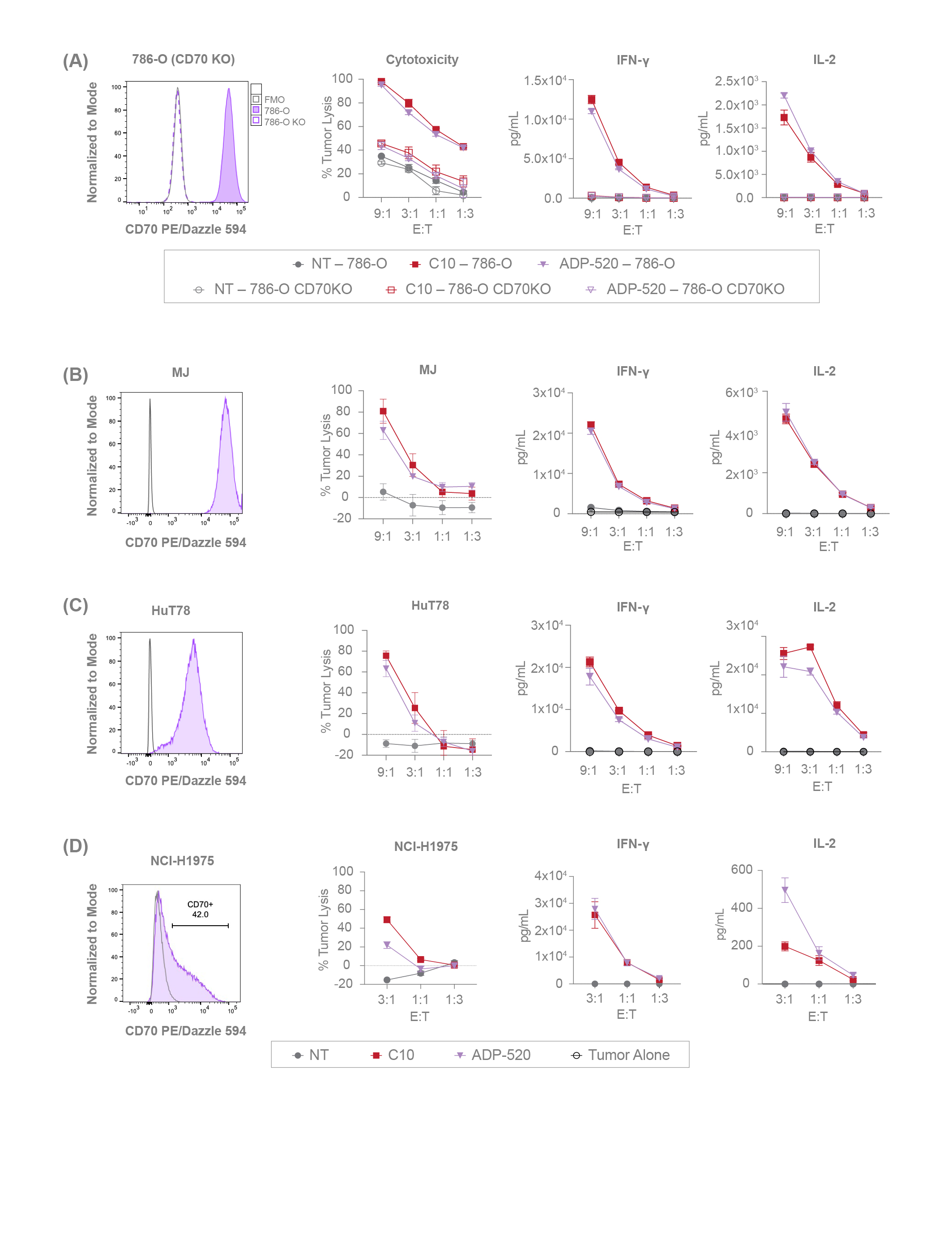

Supplement: Supplementary Figure 3 — ADP-520 TRuC T cells demonstrate in vitro efficacy against multiple CD70-expressing tumor cell lines. Titration of NT, C10 TRuC and ADP-520 TRuC T cells against tumor cell lines with different levels of CD70 expression, as demonstrated by flow cytometry histograms (shaded histogram = CD70, dashed histogram = CD70 KO, unshaded histogram = negative control). Percentages of tumor cell cytotoxicity, measured by luciferase assay, and IFN-γ and IL-2 cytokine secretion, determined by electrochemiluminescent detection, are shown for 786-O CD70 KO(A), CD70hi MJ(B), CD70mod HuT78 (C) and CD70lo NCI-H1975 (D) tumor cell lines at E:T ratios of 3:1, 1:1 and 1:3. Cytotoxicity/effector function is compared between wild-type and CD70 KO 786-O lines (A). Data represent n=2 donor batches measured in technical triplicate. E:T, effector:target; KO, knockout; PE, phycoerythrin; TRuC, T-cell receptor fusion construct. [file Image3.jpg]

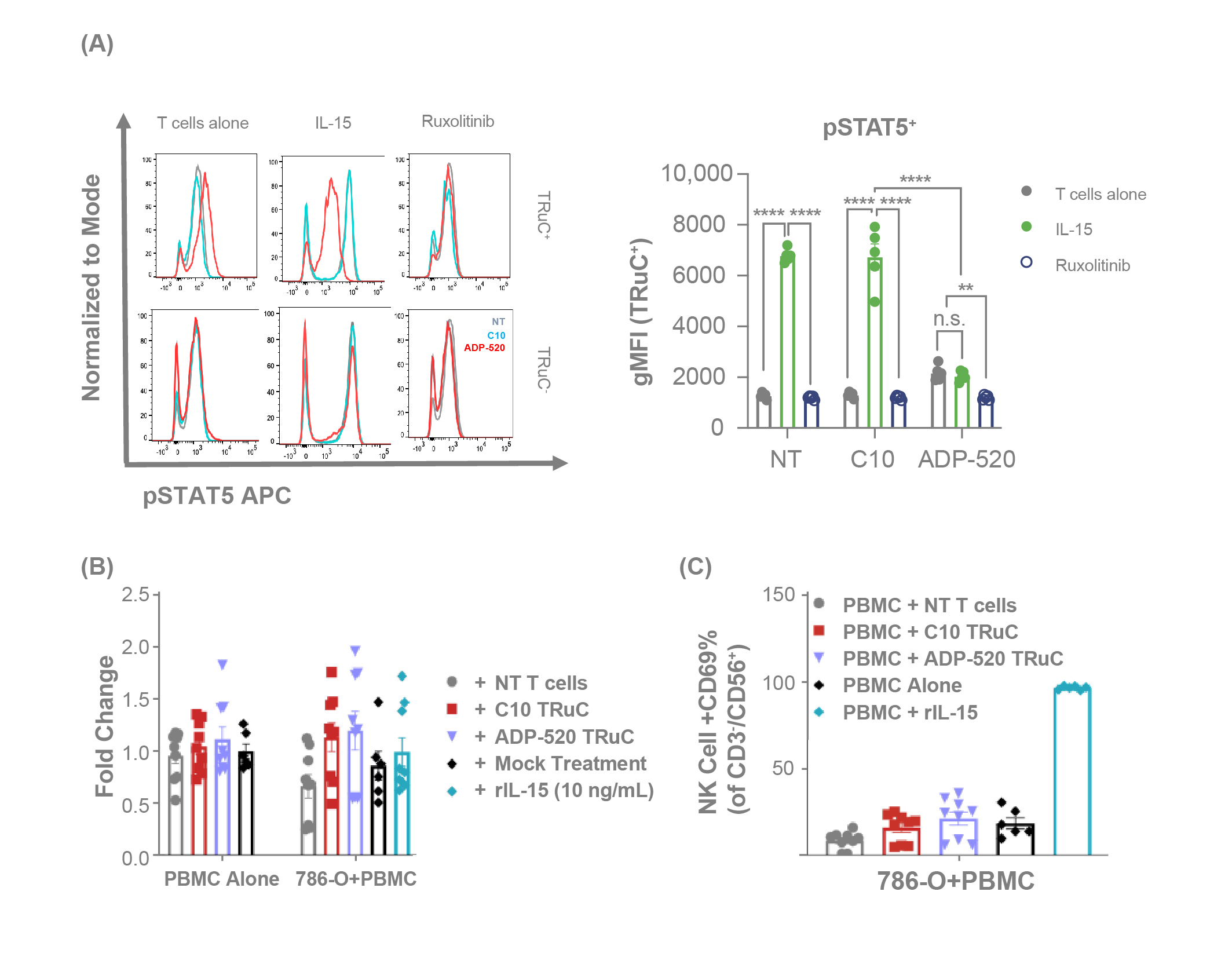

Supplement: Supplementary Figure 4 — ADP-520 TRuC T-cells are non-responsive to exogenous IL-15 stimulation and constitutive mbIL-15 expression induced negligible NK cell activation. (A) Flow cytometric analysis of pSTAT5 expression in NT, C10 TRuC and ADP-520 TRuC T cells in response to exogenous IL-15 stimulation or JAK1/2 inhibition. Histograms depict representative pSTAT5 expression in TRuC+ and TRuC- populations from a single donor, with column chart showing mean (± SEM) pSTAT5 gMFI quantification in the TRuC+ population of n=6 donors measured in technical triplicate. (B) Flow cytometric quantification bystander NK cell expansion, measured by fold-expansion at 72-hour of co-culture, relative to Hour 0, in the presence or absence of CD70+ 786-O tumor cells during 1:1 co-culture with mock treatment (no T cells), donor-matched NT T cells, C10 TRuC T cells, or ADP-520 T cells. Recombinant human IL-15 (10 ng/mL final concentration) was used as positive control to induce NK activation. (C) Frequency of CD69 positive CD3-/CD56+ NK cells measured at 24 hours after co-culture treatment. Recombinant human IL-15 (10 ng/mL final concentration) was used as positive control to induce NK activation. Data are plotted as mean ± SEM from experiments performed using at least two donor T cell batches with measurements performed in triplicate. CD, cluster of differentiation; gMFI, geometric mean fluorescence intensity; IL, interleukin; JAK, Janus kinase; mbIL-15, membrane-bound IL-15; NK, natural killer; NT, non-transduced; PBMC, peripheral blood mononuclear cell; pSTAT5, phosphorylated signal transducer and activator of transcription 5; SEM, standard error of the mean; TRuC, T-cell receptor fusion construct. [file Image4.jpg]

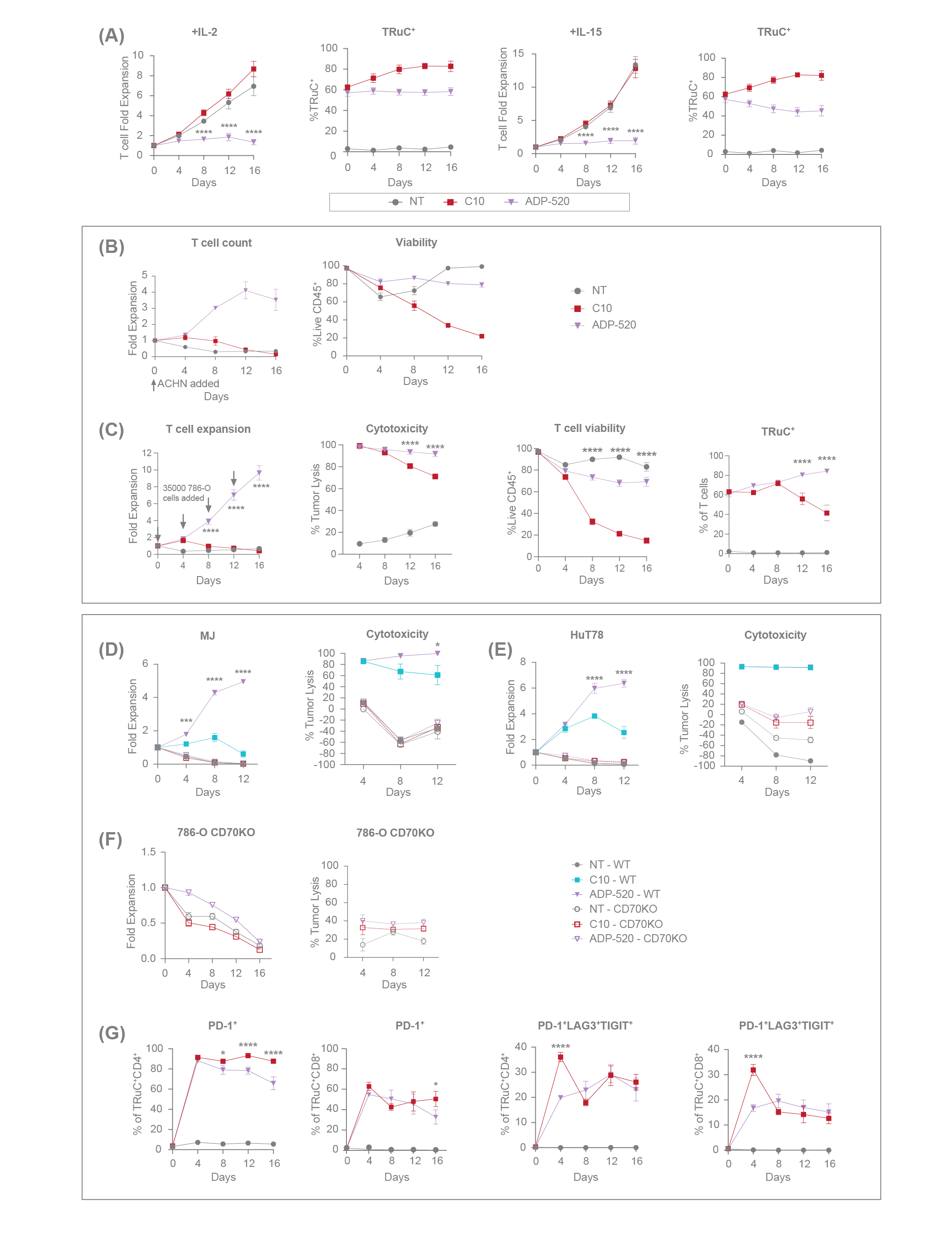

Supplement: Supplementary Figure 5 — ADP-520 cells show improved T-cell expansion, persistence and resistance to exhaustion. (A) Flow cytometric quantification of NT, C10 TRuC and ADP-520 TRuC T-cell expansion and percentage of TRuC+ cells in the presence of exogenous cytokine stimulation (IL-15 or IL-2). (B) Antigen-dependent persistence of NT, C10 TRuC and ADP-520 TRuC T cells, showing growth curves in the presence of ACHN tumor cells at a 5:1 E:T ratio and corresponding quantification of the percentage of live CD45+ T cells at each timepoint. Expansion and cytotoxicity are shown for NT, C10 TRuC and ADP-520 TRuC T cells in response to multiple challenges from 786-O (C), MJ (D), HuT78 (E) and 786-O CD70 KO tumor cells (F). Arrows indicate timepoint of additional tumor cell additions to coculture. Corresponding T-cell viability (as percentage of live CD45+ cells) and percentage of TRuC+ cells also shown for the 786-O coculture. (G) Flow cytometric quantification of T-cell exhaustion markers (PD-1 or the combination of PD-1, LAG3 and TIGIT) as a percentage of CD4+ or CD8+ NT, C10 and ADP-520 TRuC+ cells during the multiple 786-O rechallenges depicted in Supplementary Figure 5C. Data are mean ± SEM of n=3 donors, measured in technical triplicate. Treatment groups were compared via two-way ANOVA, followed by Dunnett’s multiple comparison test; *p < 0.05, **p < 0.01, ***p < 0.001, ****p < 0.0001. APC, allophycocyanin; KO, knockout; NT, non-transduced; pSTAT5, phosphorylated signal transducer and activator of transcription 5; SEM, standard error of the mean; TRuC, T-cell receptor fusion construct. [file Image5.jpg]

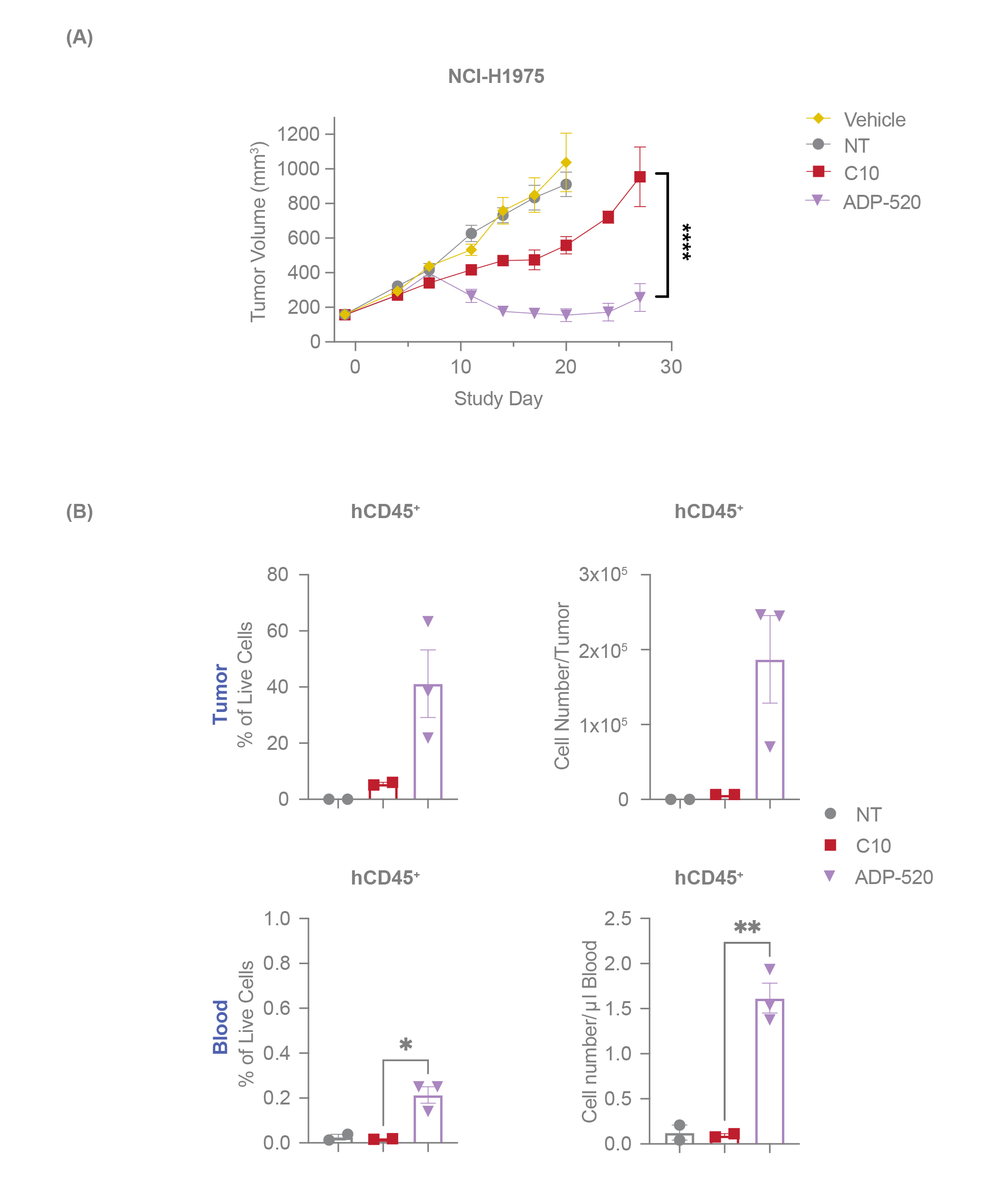

Supplement: Supplementary Figure 6 — In vivo efficacy of ADP-520 TRuC T cells in an NSCLC NCI-H1975 tumor model. (A) Tumor volume in a subcutaneous NSG MHC class I/II KO mouse xenografted with 5 x 10^6^ tumor cells/mouse and then treated after 15 days by intravenous infusion of 1.4 x 10^6^ NT T cells, or 1 x 10^6^ C10 TRuC or ADP-520 TRuC T cells. (B) Flow cytometric quantification of human vs mouse CD45 expression in the blood and tumor of the mice treated in Supplementary Figure 6A. Data presented as percentage of CD45+ live cells and number of cells per tumor or microliter of blood at 14 days post-treatment. Experiments were performed using T cells from a single representative human donor. In vivo data are mean ± SEM of n=5 mice per group, with treatment groups compared using a two-way repeated measures ANOVA, followed by the Bonferroni post hoc test. Ex vivo are mean ± SEM of n=3 mice per group, with treatment groups compared using one-way ANOVA followed by Dunnett’s multiple comparison test; *p < 0.05, **p < 0.01, ****p < 0.0001. ANOVA, analysis of the variance; KO, knockout; MHC, major histocompatibility complex; NSG, NOD SCID gamma; NT, non-transduced; SEM, standard error of the mean; TRuC, T-cell receptor fusion construct. [file Image6.jpg]
